# Molecular identification and pathogenic impact of *Pythium aphanidermatum* on ginger (*Zingiber officinale*): insights into oxidative stress, antioxidant responses, and mycotoxin profiling

**DOI:** 10.3389/fmicb.2025.1626700

**Published:** 2025-09-25

**Authors:** Mukesh Meena, Garima Yadav, Priyankaraj Sonigra, Tushar Mehta, Adhishree Nagda, Abhishek Sahoo, Prashant Swapnil

**Affiliations:** ^1^Laboratory of Phytopathology and Microbial Biotechnology, Department of Botany, Mohanlal Sukhadia University, Udaipur, Rajasthan, India; ^2^Department of Botany, School of Basic Sciences, Central University of Punjab, Bathinda, India

**Keywords:** ginger, *Pythium aphanidermatum*, oxidative stress, antioxidant enzymes, chlorophyll degradation, fungal pathogenicity, disease management

## Abstract

**Introduction:**

Ginger (*Zingiber officinale*) is an economically significant crop, widely cultivated for its medicinal and culinary applications. However, its production is severely affected by fungal pathogens, particularly *Pythium aphanidermatum*, which cause substantial yield losses and quality deterioration. This study aimed to identify fungal pathogens associated with ginger rhizome rot through molecular characterization and evaluate their impact on plant physiological and biochemical responses.

**Methods:**

Fungal isolates were obtained from infected rhizomes and identified through morphological and molecular characterization using ITS1 and ITS4 primers. Pathogenicity was evaluated via *in vitro* and *in vitro* assays, with analyses of oxidative stress and enzymatic activity. Antioxidant and phenolic metabolism enzyme activities were measured, and mycotoxin profiling was conducted using column chromatography and gas chromatography–mass spectrometry (GC–MS).

**Results:**

The results confirmed the presence of *P. aphanidermatum*, which induced severe oxidative stress in ginger plants, including increased reactive oxygen species (ROS) accumulation, lipid peroxidation, and chlorophyll degradation. Antioxidant enzymes such as ascorbate peroxidase (APX), catalase (CAT), superoxide dismutase (SOD), and glutathione reductase (GR) were significantly upregulated, along with phenylalanine ammonia-lyase (PAL) and polyphenol oxidase (PPO). Mycotoxin profiling revealed secondary metabolites contributing to fungal pathogenicity. Application of fungal crude extracts (F1–F3), 24 h prior to inoculation, significantly reduced oxidative damage and preserved plant physiological integrity, with F1 showing the most effective mitigation.

**Discussion and conclusion:**

This study demonstrates that *P. aphanidermatum* infection imposes severe oxidative stress and physiological damage in ginger, as evidenced by elevated ROS, malondialdehyde (MDA), and disrupted chlorophyll composition. Pre-application of fungal crude extracts alleviated these effects, highlighting their potential role in plant defense. These findings provide new insights into the pathogenic mechanisms of *P. aphanidermatum* and the phytotoxicity of its metabolites, laying the foundation for future studies on detailed chemical characterization and field validation.

## 1 Introduction

Ginger (*Zingiber officinale*) is a widely cultivated medicinal and culinary crop valued for its aromatic rhizomes, which contain bioactive compounds with notable antimicrobial, anti-inflammatory, and antioxidant properties (Zhukovets and Özcan, 2020). Despite its economic and therapeutic importance, ginger cultivation is severely affected by soil-borne pathogens, particularly oomycetes like *Pythium aphanidermatum*, which cause soft rot disease. In addition to *P. aphanidermatum,* other oomycetes such as *Pythium myriotylum* have also been reported as frequent causal agents of soft rot in ginger, particularly in China and Southeast Asia. Recent molecular studies confirmed *P. myriotylum* as one of the most commonly recovered pathogens from soft-rot-infected ginger rhizomes (Lv et al., 2020). These findings emphasize the broader relevance of the *Pythium* genus in ginger pathology and the need for integrative approaches to managing this pathogen group. Accurate identification of the causative pathogen is essential for effective disease management (Archana et al., 2024). Traditional morphological techniques are often insufficient to distinguish closely related species, necessitating the use of molecular tools. The internal transcribed spacer (ITS) region of ribosomal DNA (rDNA) is widely used for the identification of fungi and oomycetes due to its high inter-species variability and the availability of curated sequence databases (Yadav et al., 2023; Gahagan et al., 2023). Recent research has shown that bioactive volatile organic compounds (VOCs) emitted by *Phytophthora* and *Pythium* species can serve as non-invasive biomarkers for disease detection in agricultural systems (Sheikh et al., 2024). Such studies suggest new avenues for early diagnosis and field-based monitoring, particularly where conventional symptoms are not immediately apparent. Beyond identification, understanding the physiological and biochemical responses of infected plants is vital. Pathogen invasion triggers excessive production of reactive oxygen species (ROS), leading to oxidative stress, membrane damage, and disruption of cellular processes. In response, plants activate their antioxidant defense systems, particularly enzymes such as catalase (CAT), superoxide dismutase (SOD), and ascorbate peroxidase (APX), which help mitigate oxidative damage (Fujita and Hasanuzzaman, 2022; González-Guzmán et al., 2020). In addition to enzymatic degradation of plant tissue, *P. aphanidermatum* is known to secrete toxic secondary metabolites, which exacerbate host tissue necrosis and increase disease severity. Profiling these metabolites using chromatographic and mass spectrometric methods is critical to understanding the pathogen’s virulence mechanisms (Mapuranga et al., 2022; Kumar et al., 2024). The impact of soft rot is especially devastating in regions where monoculture practices, inadequate soil drainage, and traditional farming methods create favorable environments for pathogen proliferation. These pathogens persist in soil and can survive as spores, making disease control difficult (Huang et al., 2024; Parveen and Sharma, 2015). Infected plants typically show symptoms such as chlorosis, necrosis, water-soaked lesions, wilting, and rhizome decay ultimately leading to significant yield and quality losses. *P. aphanidermatum* thrives under warm and humid conditions and is characterized by its rapid infection cycle facilitated by the production of motile zoospores, which actively target host roots for penetration. Its virulence is mediated by extracellular enzymes like cellulases and pectinases that degrade plant cell walls, as well as by phytotoxic metabolites that interfere with host cellular integrity (Xu et al., 2021; Chen et al., 2022). These factors collectively induce oxidative stress in the host, impairing redox homeostasis and contributing to cell death.

Although antioxidant enzymes such as SOD, CAT, APX, and glutathione reductase (GR) are known to be upregulated in response to pathogen attacks (Kaur et al., 2022; Gong et al., 2023), their specific roles in ginger’s defense against *P. aphanidermatum* remain poorly understood. Furthermore, while some studies have documented mycotoxin production by *Pythium* sp., the chemical nature and physiological impacts of these metabolites in ginger are still largely unexplored. Disease-induced oxidative stress also leads to a decline in photosynthetic efficiency, chlorophyll content, and overall plant biomass (Berger et al., 2007; Fan and Li, 2024; Wang et al., 2024), severely compromising crop productivity and market value. The use of PCR-based molecular methods, especially ITS sequencing with universal primers ITS1 and ITS4, has become a standard approach for accurate and rapid pathogen identification (Levesque and De Cock, 2004; Alsharksi et al., 2024; Zhang et al., 2024). Coupling molecular diagnostics with an understanding of host physiological responses and pathogen metabolite profiling provides a comprehensive framework for disease management. The present study aims to comprehensively investigate the pathogenesis of *P. aphanidermatum* in ginger (*Zingiber officinale*) by employing a multi-faceted approach. Specifically, the objectives include: (i) accurate identification and characterization of the pathogen using ITS region sequencing and morphological analysis; (ii) evaluation of the physiological and oxidative stress responses in ginger plants triggered by fungal infection, with emphasis on key antioxidant enzymes such as SOD, CAT, and APX; and (iii) profiling of secondary metabolites produced by the pathogen to understand their role in disease progression. The significance of this research lies in its potential to provide critical insights into host–pathogen interactions at molecular and biochemical levels, which are essential for devising effective and sustainable disease management strategies. The outcomes are expected to support the development of resistant ginger cultivars, identify potential targets for biocontrol interventions, and inform agronomic practices that can mitigate the impact of soil-borne fungal diseases, thereby safeguarding crop productivity and farmer livelihoods.

## 2 Materials and methods

### 2.1 Sample collection and fungal isolation

Ginger (*Zingiber officinale*) rhizomes showing symptoms of rot, discoloration, and wilting were collected from multiple agricultural fields in Udaipur, and nearby areas such as Jhadol, Gogunda, Badgaon, etc. The samples were obtained from farms with a history of fungal infections and were transported to the laboratory under sterile conditions. To ensure a diverse collection, samples were taken from plants at different growth stages and from different soil types. The diseased rhizomes were carefully cleaned to remove soil debris, and symptomatic tissue sections were excised for further analysis. Fungal pathogens were isolated using the tissue segment method. Small infected rhizome sections (5 × 5 mm) were surface sterilized with 1% sodium hypochlorite (NaOCl) for 1–2 min, followed by rinsing with sterile distilled water to remove residual disinfectants. The sterilized tissue sections were plated onto Potato Dextrose Agar (PDA) medium supplemented with 50 mg/L streptomycin to prevent bacterial contamination. The plates were incubated at 25 ± 2 °C for 5–7 days, and fungal growth was monitored daily. Emerging fungal colonies were sub-cultured onto fresh PDA plates to obtain pure cultures. Morphological characteristics such as colony color, texture, conidial morphology, and growth patterns were recorded using a light microscope.

### 2.2 Molecular identification of fungal isolates

#### 2.2.1 DNA extraction and PCR amplification

Genomic DNA was extracted from pure fungal cultures using the cetyltrimethylammonium bromide (CTAB) method. Fungal mycelia were harvested from 7-day-old cultures grown in liquid potato dextrose broth (PDB) and ground into a fine powder using liquid nitrogen. The powdered mycelial mass was mixed with CTAB extraction buffer (100 mM Tris–HCl, 1.4 M NaCl, 20 mM EDTA, 2% CTAB) and incubated at 65 °C for 30 min. The DNA was purified by phenol-chloroform extraction and precipitated using absolute ethanol. The DNA pellets were resuspended in TE buffer (10 mM Tris–HCl, 1 mM EDTA) and stored at −20 °C for further use. Polymerase chain reaction (PCR) amplification of the internal transcribed spacer (ITS) region of ribosomal DNA was performed using universal primers ITS1 (5’-TCCGTAGGTGAACCTGCGG-3′) and ITS4 (5’-TCCTCCGCTTATTGATATGC-3′). The PCR reaction mixture (25 μL) contained 2.5 μL of 10 × PCR buffer, 2 μL of 2.5 mM dNTP mix, 1.5 μL of 25 mM MgCl₂, 1 μL of each primer (10 μM), 0.5 μL of Taq DNA polymerase (5 U/μL), and 2 μL of template DNA. The amplification was carried out in a thermal cycler under the following conditions: initial denaturation at 95 °C for 3 min, followed by 35 cycles of denaturation at 95 °C for 30 s, annealing at 56 °C for 30 s, and extension at 72 °C for 1 min, with a final extension at 72 °C for 10 min. PCR products were analyzed by electrophoresis on a 1.5% agarose gel stained with ethidium bromide and visualized under a UV transilluminator. Successful amplifications resulted in DNA fragments of approximately 500–600 bp (Meena et al., 2016). The amplified products were purified using a PCR purification kit and sent for sequencing. The obtained sequences were compared with reference sequences in the National Center for Biotechnology Information (NCBI) GenBank database using the BLAST algorithm for species identification.

### 2.3 Pathogenicity testing

The pathogenic potential of the isolated fungal species was assessed using both *in vivo* and *in vitro* assays.

#### 2.3.1 *In vivo* pathogenicity assay

Following the protocol of Le et al. (2014), healthy ginger plants were cultivated in pots containing sterilized soil and maintained under greenhouse conditions at 25 ± 2 °C with approximately 70% relative humidity. To assess pathogenicity, a wound inoculation technique was employed. Fungal spore suspensions (1 × 10^6^ spores/mL), prepared from 7-day-old cultures grown in potato dextrose broth (PDB), were used for inoculation. Small wounds (~2–3 mm deep and ~1–2 mm in diameter) were introduced into the central portion of each rhizome using a sterile needle. A volume of 100 μL of the fungal spore suspension (1 × 10^6^ spores/mL) was applied directly to the wound site. Each treatment group (including control) consisted of five biological replicates (*n* = 5), and the entire experiment was repeated three times independently to ensure reproducibility. Control plants were mock-inoculated with sterile distilled water or sterile PDB medium, and maintained under identical greenhouse conditions to assess background physiological responses. All treated plants were observed over a 14-day period to monitor symptom development, and disease severity was evaluated using a visual scale based on lesion dimensions and the extent of rhizome decay.

#### 2.3.2 *In vitro* leaf bioassay

To further validate pathogen virulence, detached leaf assays were performed following the method described by Farinas et al. (2019). Fresh ginger leaves were collected and placed onto moist filter papers inside sterile Petri dishes. A volume of 10 μL fungal spore suspension was applied directly onto the surface of each leaf. The inoculated plates were incubated at 25 °C, and the development of lesions was monitored after 72 h. Virulence assessment was based on calculating the percentage of leaf area exhibiting infection symptoms.

### 2.4 *In vitro* production of the metabolite from the pathogen

The *in vitro* method used to obtain metabolites from pathogens was adapted from Mutawila et al. (2016). In the current investigation, a single virulent isolate of *P. aphanidermatum* was cultured in triplicate under controlled *in vitro* conditions for the extraction of toxic metabolites. Metabolite production occurred primarily using potato dextrose broth (PDB), supplemented by another chemically-defined culture medium composed of glucose (40 g/L), KH₂PO₄ (1.0 g/L), (NH₄)₂HPO₄ (2.0 g/L), MgSO₄·7H₂O (0.5 g/L), KCl (0.5 g/L), and yeast extract (1.0 g/L). After dissolving these components in one liter of distilled water, the pH was adjusted to 5.5. Subsequently, 200 mL of the prepared medium was dispensed into 500 mL flasks and sterilized and the isolate was inoculated.

Each flask was inoculated with 1 mL of fungal spore suspension, standardized at a concentration of approximately 3 × 10^5^ spores per ml. The cultures were incubated at 25 °C for 25 days. Following incubation, fungal biomass was removed by filtering the culture medium sequentially through glass wool, Whatman No. 1 filter paper, and ultimately a sterile syringe filter with a pore size of 0.22 μm. The resulting filtrate, enriched with fungal metabolites, was refrigerated at 4 °C for subsequent assays. Uninoculated sterile medium served as the experimental control for comparison in all conducted experiments (Meena et al., 2017a,b).

### 2.5 Extraction of the toxic metabolites from fungal isolates

Extractions of the secondary metabolites have been carried out according to the method of Frisvad et al. (2008). The extractions of these phytotoxins have been carried out on PDB medium by using 20-day old cultures. The sterile PDB medium was inoculated with 1 mL of spore suspension containing 3 × 10^5^ spores/ml and incubated for 25 days at 25 °C. The culture filtrate was then collected by successive passage through glass wool, Whatman No.1 filter paper, and finally through a syringe filter (0.22 μm). These filtrates containing the metabolite(s) were stored at 4 °C until further use. An uninoculated medium served as a control in all the experiments.

### 2.6 Purification and separation of *Pythium* phytotoxins via column chromatography

Purification and separation of toxic compounds from the crude extract of *P. aphanidermatum* were carried out following the methodology described by Devi et al. (2023), with minor modifications. Column chromatography (CC) was performed using a glass column (700 mm × 30 mm) packed with silica gel (100–120 mesh size, Merck) as the stationary phase. The column was loaded with the crude fungal extract, and elution was carried out using solvent systems optimized for compound separation. A gradient elution technique was applied using chloroform:methanol mixtures (95:5 and 80:20, v/v), followed by a ternary solvent system comprising benzene:acetone:acetic acid in a 60:35:5 ratio. These fractions will subsequently be subjected to toxicity assessment to evaluate their potential biological effects (Meena et al., 2017a,b).

### 2.7 Toxicity assay

To evaluate the phytotoxicity of the fractions obtained from the crude extract of *P. aphanidermatum*, an *in vitro* leaf bioassay was conducted. Each fraction (3 mL) was uniformly applied to a 7 cm diameter Whatman No.1 filter paper placed at the base of a sterile Petri dish. A fully expanded, healthy leaf was positioned with its abaxial (lower) surface in direct contact with the moistened filter paper. A small incision was carefully made along the midrib on the lower side of the leaf to facilitate the uptake of the test solution into the leaf tissues. The Petri dishes were sealed and incubated under continuous illumination at 25 ± 1 °C for a period of six days. Symptoms of leaf necrosis were observed and recorded at 24 h intervals. As controls, leaves were placed on filter papers moistened either with sterile deionized water or with uninoculated PDB medium to differentiate between physiological effects caused by fungal metabolites and baseline responses to the culture medium.

### 2.8 Cell death assay using Evans blue staining

The degree and extent of cell death were determined by Evans blue staining, as described by Jacyn Baker and Mock (1994). For this method, plants were infected with the pathogen and challenged with metabolites. After 48 h, the affected leaves were boiled for 1–2 min in a freshly prepared solution of phenol: lactic acid: glycerol: distilled water (1:1:1:1) containing 20 mg/mL Evans blue stain. The tissues were then clarified overnight in a solution of 2.5 g/mL chloral hydrate in water. Cell death was observed under a light microscope.

### 2.9 DAB staining for hydrogen peroxide

Hydrogen peroxide (H₂O₂) levels were determined through histochemical staining using diaminobenzidine (DAB) following the method outlined by Thordal-Christensen et al. (1997). For the procedure, the plants were initially exposed to the pathogen and its related metabolites. After 48 h of infection, leaves were carefully excised with a sterilized blade just above the petiole base and immersed in a solution containing 1 mg/mL of 3,3′-diaminobenzidine hydrochloride (DAB-HCl) at pH 5.6. The treated leaves were then placed in a moist growth chamber and incubated in the dark overnight (approximately 12 h). Hydrogen peroxide present within the tissue interacts with DAB, resulting in a reddish-brown coloration. Subsequently, the chlorophyll was removed from the samples using 96% boiled ethanol, enabling clear visualization of H₂O₂ accumulation under a light microscope.

### 2.10 Estimation of chlorophyll content

In ginger plants, disease development was also assessed by observing the chlorophyll content (Chl a, Chl b, and total chlorophyll). In this method, tomato plant leaves (0.1 g) that were infected with the pathogen and treated with the toxic metabolites of *Pythium* sp. were chopped into small pieces and extracted with 80% acetone. Chlorophyll contents were estimated by measuring the absorbance at 645 nm and 663 nm for chlorophyll a, b, and total chlorophyll. Then chlorophyll a, b, and total chlorophyll were further calculated according to the Lichtenthaler and Wellburn (1983) formulae:


$$\mathrm{Chl}\;a\;(\frac{\mathrm{mg}}{g}\text{leaf fresh weight})=\frac{\left[\begin{array}{l} (12.7\times {\mathrm{OD}}_{663})-\\ \left(2.69\times {\mathrm{OD}}_{645}\right) \end{array}\right]}{\left(1000\times W\right)}\times V$$

$$\mathrm{Chl}\;b\;(\frac{\mathrm{mg}}{g}\text{leaf fresh weight})=\frac{\left[\begin{array}{l} (22.9\times {\mathrm{OD}}_{625})-\\ \left(4.68\times {\mathrm{OD}}_{663}\right) \end{array}\right]}{\left(1000\times W\right)}\times V$$

$$\text{Total}\;\mathrm{Chl}\;(\frac{\mathrm{mg}}{g}\text{leaf fresh weight})=\frac{\left[\begin{array}{l} (20.2\times {\mathrm{OD}}_{645})+\\ \left(8.02\times {\mathrm{OD}}_{663}\right) \end{array}\right]}{\left(1000\times W\right)}\times V$$

Where OD = Optical Density, V = Volume of the sample, and W = Weight of the sample.

### 2.11 Biochemical analysis

#### 2.11.1 Oxidative stress markers

Oxidative stress in infected plants was evaluated by measuring hydrogen peroxide (H₂O₂) accumulation and lipid peroxidation.

##### 2.11.1.1 H₂O₂ quantification

Hydrogen peroxide accumulation was assessed following the method described by Thordal-Christensen et al. (1997). Leaf tissues were incubated in a 1 mg/mL solution of 3,3′-diaminobenzidine (DAB) for 8 h in the dark. After incubation, leaves were cleared with ethanol to remove chlorophyll, enhancing the visibility of brown precipitates formed by the reaction between DAB and hydrogen peroxide. The extent of staining, indicative of H₂O₂ presence, was quantified spectrophotometrically by measuring absorbance at 390 nm.

##### 2.11.1.2 Lipid peroxidation

Lipid peroxidation was estimated following the procedure outlined by Ohkawa et al. (1979), which quantifies malondialdehyde (MDA), a byproduct of polyunsaturated fatty acid oxidation. Leaf tissues (0.1 g) were homogenized in 2.0 mL of 20% trichloroacetic acid (TCA) containing 1% thiobarbituric acid (TBA). The mixture was incubated at 95 °C for 30 min. To stop the reaction, samples were cooled on ice for 10 min and then centrifuged at 10,000 rpm for 15 min. The absorbance of the supernatant was recorded at 532 nm, and MDA content was expressed as μmol MDA per gram of fresh weight.

#### 2.11.2 Antioxidant enzyme activities

The activity of antioxidant enzymes was assessed in infected and control plant tissues.

##### 2.11.2.1 Superoxide dismutase

SOD activity was determined based on its capacity to inhibit the photoreduction of nitro blue tetrazolium (NBT), following the method described by Flohé (1984). Tomato leaves (0.1 g) were homogenized in 5 mL of ice-cold extraction buffer composed of 0.1 M phosphate buffer (pH 7.5) and 0.5 mM EDTA. The homogenate was centrifuged at 15,000 rpm for 15 min, and the supernatant was used as the enzyme extract. The reaction mixture (3 mL) contained 50 mM phosphate buffer (pH 7.8), 13 mM methionine, 75 μM NBT, 60 μM riboflavin, 0.1 mM EDTA, and 100 μL of the enzyme extract. Reactions were incubated at 25 °C under fluorescent light for 10 min. SOD activity was expressed in units, where one unit corresponds to the amount of enzyme needed to cause 50% inhibition of NBT reduction, as measured by absorbance at 560 nm.

##### 2.11.2.2 Catalase

Catalase activity was determined according to the method described by Aebi (1984). Leaf samples (0.1 g) were ground in a chilled mortar and pestle using 5 mL of extraction buffer consisting of 50 mM Tris–HCl (pH 8.0), 0.5 mM EDTA, 2% (w/v) polyvinylpyrrolidone (PVP), and 0.5% (v/v) Triton X-100. The homogenate was centrifuged at 15,000 rpm for 10 min at 4 °C, and the resulting supernatant was used as the enzyme extract. For the assay, 1 mL of enzyme extract was mixed with 300 μM phosphate buffer (pH 7.2) containing 100 μM hydrogen peroxide (H₂O₂). The decomposition of H₂O₂ was monitored in the dark for 1 min by measuring the decline in absorbance at 240 nm. Catalase activity was calculated based on the amount of H₂O₂ broken down and expressed as nmol of H₂O₂ decomposed per minute per gram of fresh weight.

##### 2.11.2.3 Ascorbate peroxidase

APX activity was measured following the protocol of Nakano and Asada (1987). The assay mixture contained 0.2 mL of enzyme extract, 25 mM phosphate buffer (pH 7.0), 0.1 mM EDTA, 0.25 mM ascorbic acid, and 1.0 mM H₂O₂. The reaction was initiated by adding the enzyme extract, and the decrease in absorbance was monitored at 290 nm after 60 s. Enzymatic activity was calculated based on the rate of ascorbate oxidation and expressed as nmol ascorbate oxidized per minute per mg of protein.

##### 2.11.2.4 Glutathione reductase

GR activity was assayed according to the method described by Yannarelli et al. (2007). Leaf tissues (0.1 g) were homogenized in 5 mL of 50 mM Tris–HCl buffer (pH 7.6) using a chilled mortar and pestle. The homogenate was centrifuged at 15,000 rpm for 30 min at 4 °C, and the supernatant was used for enzymatic analysis. The assay mixture included 50 mM Tris–HCl buffer (pH 7.6), 10 μL NADPH (0.15 mM), 100 μL oxidized glutathione (1 mM GSSG), 3 mM MgCl₂, and 0.3 mL enzyme extract. GR activity was monitored by the decrease in absorbance of NADPH at 340 nm and expressed as μmol NADPH oxidized per minute per mg of protein.

##### 2.11.2.5 Phenylalanine ammonia-lyase

Phenylalanine ammonia-lyase (PAL) activity was assessed using 0.3 g of leaf tissue collected from tomato plants exposed to pathogen and their metabolic products. The tissue was homogenized in 6.5 mL of 50 mM Tris–HCl buffer (pH 8.8) supplemented with 15 mM *β*-mercaptoethanol, using an ice-chilled mortar and pestle for approximately 5 min. The resulting homogenate was centrifuged for 30 min, after which the supernatant was recovered for the enzyme assay. PAL activity was quantified by monitoring the formation of cinnamic acid, following the protocol outlined by Ochoa-Alejo and Gomez-Peralta (1993). For the assay, a mixture containing 1 mL of extraction buffer, 0.5 mL of 10 mM L-phenylalanine, 0.4 mL deionized water, and 0.1 mL enzyme extract was incubated at 37 °C for 1 h. The reaction was halted by adding 0.5 mL of ethyl acetate, and the solvent was then evaporated to eliminate the extracting agent. The remaining solid was dissolved in 3 mL of 0.05 M NaOH, and the cinnamic acid concentration was determined spectrophotometrically by measuring absorbance at 290 nm. One unit of PAL activity was defined as the amount of enzyme catalyzing the formation of 1 μmol of cinnamic acid per minute (Wang et al., 2006).

##### 2.11.2.6 Polyphenol oxidase

Polyphenol oxidase (PPO) activity was evaluated following the procedure outlined by Mayer et al. (1965). For this assay, 1.0 g of leaf tissue was homogenized in 2 mL of 0.1 M sodium phosphate buffer (pH 6.5), then centrifuged at 16,000 rpm for 15 min at 4 °C. The resulting supernatant served as the enzyme extract. The assay reaction was prepared by mixing 200 μL of the enzyme extract with 1.5 mL of 0.1 M sodium phosphate buffer (pH 6.5). The enzymatic reaction was initiated by adding 200 μL of 0.01 M catechol substrate. PPO activity was subsequently measured by monitoring the increase in absorbance at 495 nm, with results expressed as the change in absorbance per minute per milligram of protein.

#### 2.11.3 Mycotoxin profiling

Fungal secondary metabolites were analyzed using thin-layer chromatography (TLC) (Supplementary Figure 6) and gas chromatography–mass spectrometry (GC–MS).

##### 2.11.3.1 GC–MS analysis

Among the ten column fractions (F1–F10), F1 was selected for GC–MS analysis based on its highest necrotic index in toxicity assays. The remaining fractions were retained for future analysis, and preliminary testing showed lower or moderate phytotoxicity. The chemical composition of the potential fraction of fungal crude extract of *P. aphanidermatum* was examined using a Thermo Fisher Scientific TRACE™ 1,300 gas chromatograph paired with a TSQ 9000 triple quadrupole mass spectrometer. For compound separation, two capillary columns were employed: a front TG-SQC column (15 meters in length, 0.25 mm internal diameter, 0.25 μm film thickness) and a back TG-1MS column (30 meters in length, 0.25 mm internal diameter, 0.25 μm film thickness), both made of 100% dimethyl polysiloxane. The oven temperature program began at 60 °C, holding steady for 10 min before ramping up to 250 °C, where it was held for a further 15 min. Samples of the extracted metabolites, previously diluted at a 1:100 ratio in GC–MS grade methanol, were injected using an AI 1310 autosampler operating in split mode with a 1:50 split ratio. The carrier gas was helium, flowing at a constant rate of 1.0 mL/min. Ionization was achieved at 70 eV, with the ion source and transfer line temperatures set at 250 °C and 300 °C, respectively. The extracted metabolites were analyzed using GC–MS to identify volatile compounds contributing to pathogenicity. Compound identification relied on a combination of flame ionization detection (FID) responses, retention times, peak areas, and relative abundances. The retention time and mass spectra were matched with reference libraries, including the Wiley 7 mass spectral database. Additionally, retention indices were determined using a C9–C40 n-alkane series and cross-referenced with entries in the NIST database and relevant literature (de Alencar Filho et al., 2017). Component concentrations were averaged based on peak areas from both GC and GC–MS data, and all processing was performed using Thermo Scientific™ Dionex™ Chromeleon™ software (version 7.3).

### 2.12 Statistical analysis

All data were expressed as mean ± standard deviation (SD) from three biological replicates. One-way analysis of variance (ANOVA) was performed using OriginPro 2025 to determine statistical differences among treatment groups, followed by Tukey’s HSD test for multiple comparisons. A *p*-value < 0.05 was considered statistically significant. All biochemical assays were conducted in triplicate, and values are presented as mean ± standard deviation (SD)Tukey’s test was applied for *post hoc* multiple comparisons. Significance was determined at *p* < 0.05 for all biochemical and physiological parameters. Analyses covered enzyme activities (SOD, CAT, APX, GR, PAL and PPO), oxidative stress markers (H_2_O_2_, MDA), and chlorophyll content. Graphs with error bars representing SD were generated to visualize treatment effects over time. All statistical analyses and visualizations were performed using OriginPro 2025. All the experiments were performed in triplicate.

## 3 Results

### 3.1 Survey and disease symptom observation

**Figure 1:**
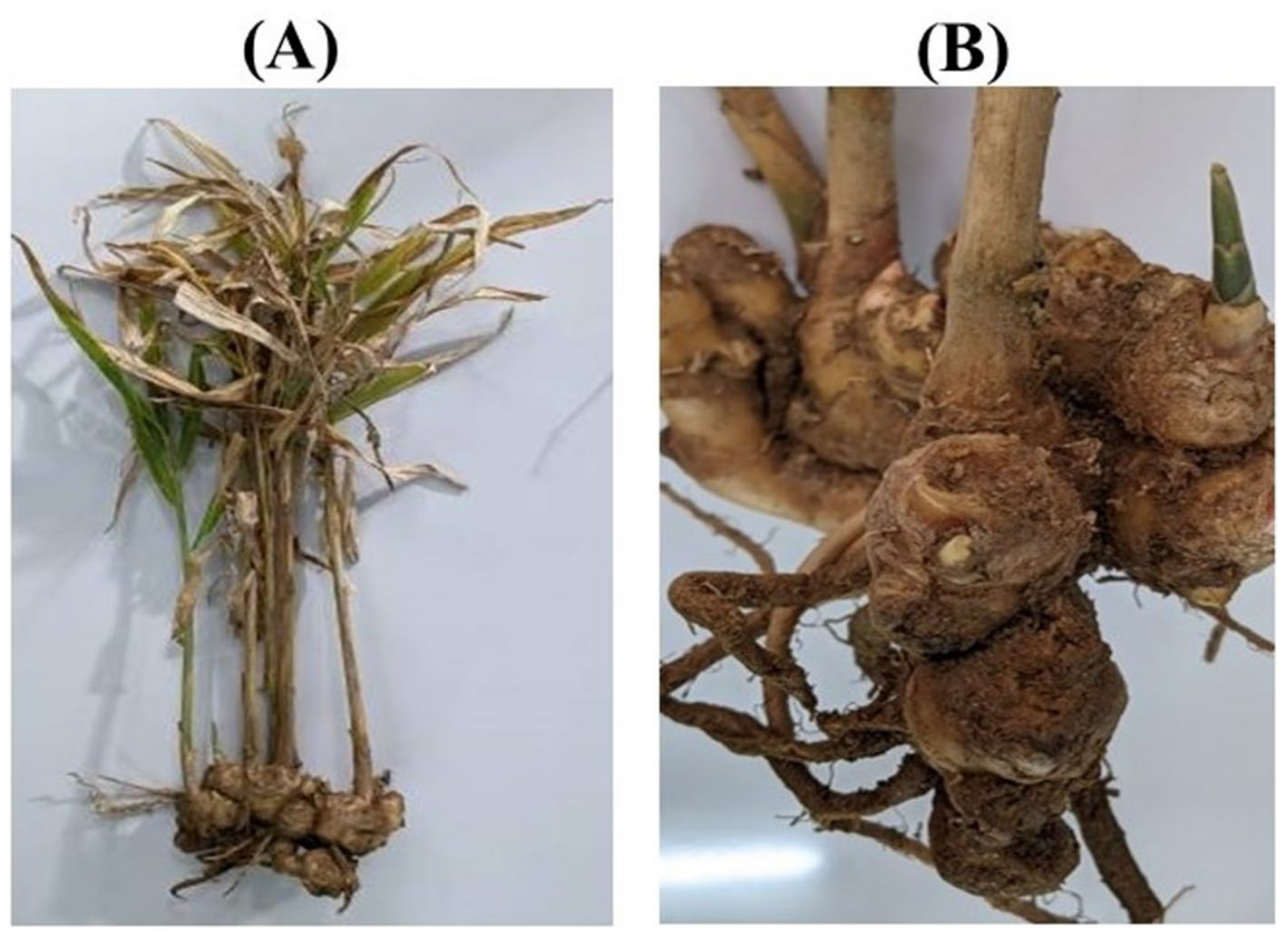
Observation of disease symptoms of *P. aphanidermatum* on ginger plants in the field; **(A)** Whole plant, and **(B)** Rhizomes.

A systematic field survey was conducted across multiple locations in Udaipur to identify and document disease symptoms in ginger plants. Observations revealed yellowing of older leaves, necrotic lesions, and rotting rhizomes, indicative of fungal infections (Supplementary Figure 1). The symptomatic plants showed a progressive decline in health, with leaves exhibiting chlorosis and necrosis, and rhizomes turning soft and brown. The infected rhizomes exhibited a foul odor, suggesting secondary microbial infections. Disease symptoms were more prevalent in waterlogged fields and poorly drained soils, highlighting the role of environmental factors in disease incidence. The collected samples were further analyzed in the laboratory to confirm the presence of fungal pathogens. Images of affected plant parts, including leaves, stems, and rhizomes, were captured to document disease progression (Figure 1).

### 3.2 Isolation and morphological identification of fungal pathogens

Fungal species were isolated from infected plant tissues using potato dextrose agar (PDA) medium. Microscopic examinations identified the presence of *P. aphanidermatum*. The isolates were further confirmed through molecular identification techniques. The colony morphology and sporulation patterns were compared with standard taxonomic descriptions to ensure accurate species identification (Supplementary Figure 2).

### 3.3 Molecular identification of pathogens

Molecular identification was performed using PCR amplification of the ITS region with ITS1 and ITS4 primers. The amplified DNA fragments (~500–600 bp) were sequenced and compared with sequences in the NCBI GenBank database (Supplementary Figure 3). BLAST analysis confirmed the identity of *P. aphanidermatum* (OP394047) as the primary pathogens responsible for ginger rhizome rot. The presence of this fungal species in multiple samples indicated their widespread distribution and significant role in disease etiology. A phylogenetic tree was constructed using ITS sequences of related *Pythium* species. The isolate clustered closely with *P. aphanidermatum* and *P. insidiosum*, supported by a bootstrap value of 65% (Supplementary Figure 4), confirming its identity as *P. aphanidermatum*.

### 3.4 Pathogenicity testing

Pathogenicity assays were performed on healthy ginger plants using wound inoculation and detached leaf assays. Infected plants developed typical disease symptoms within 14 days, including wilting, chlorosis, and rhizome decay. The severity of symptoms was more pronounced in *Pyth*ium-infected plants, which exhibited rapid disease progression. Control plants remained healthy, confirming the pathogenic role of *P. aphanidermatum*. The detached leaf assays showed necrotic spots and tissue collapse in infected leaves, whereas control leaves remained unaffected.

### 3.5 Evaluation of the toxicological efficacy of fractions derived from *Pythium* crude extract

The phytotoxic effects of the three distinct fractions isolated from the crude extract of *P. aphanidermatum* were evaluated based on their ability to induce tissue damage in tomato leaves. Notably, the intensity and progression of cellular damage varied significantly among the fractions, suggesting differential toxic potential of the constituent mycotoxins. Each fraction was independently applied to separate tomato leaf samples, and the resulting necrotic lesions were monitored over 6 days to assess the extent of tissue damage (Supplementary Figure 5). The severity of phytotoxicity was quantified by calculating the percentage of the leaf surface area exhibiting necrosis. Among all fractions, F1 consistently exhibited the highest necrotic effect across all 5 days, with a progressive increase in necrosis area from approximately 42% on day 1 to 96% on day 5. This suggests a potent phytotoxic effect, making F1 the most virulent fraction in this study. F2 and F3 also showed substantial toxicity, with necrosis areas reaching around 85–90% by day 5, though slightly lower than F1. Moderate toxicity was observed in fractions F4 to F6, with necrotic areas ranging from approximately 20–65% over the observation period. Fractions F7 to F10 demonstrated the least phytotoxicity, with final necrosis percentages staying below 30%, indicating minimal damage and a relatively weak toxic effect on leaf tissues.

### 3.6 Cell death assay by Evans blue staining

**Figure 2:**
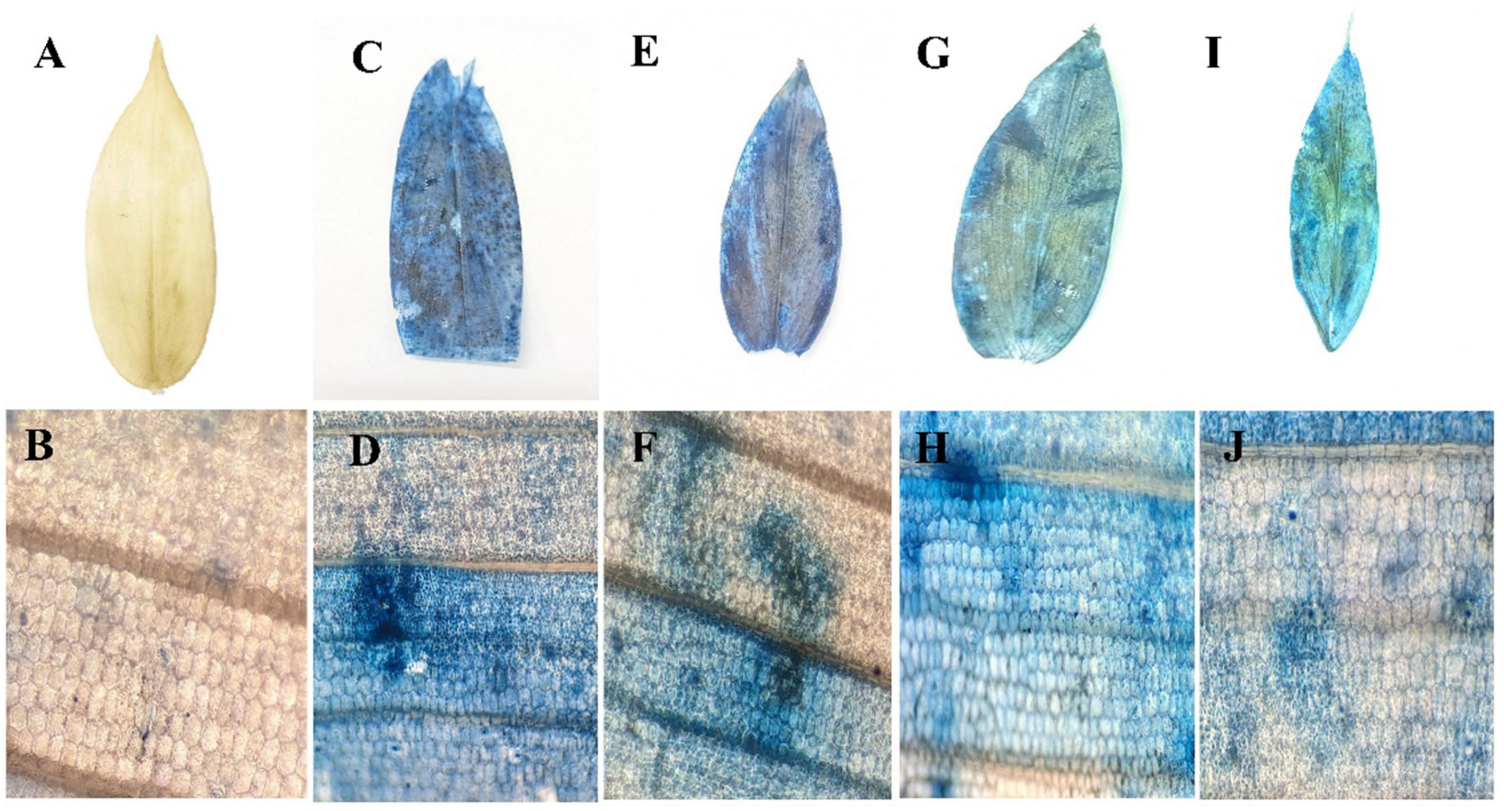
Cell death assay by Evans blue staining. **(A)** control leaf; **(B)** microscopic view; **(C)** *P. aphanidermatum* treated leaf; **(D)** microscopic view; **(E)** fraction 1 treated leaf; **(F)** microscopic view; **(G)** fraction 2 treated leaf; **(H)** microscopic view; **(I)** fraction 3 treated leaf; **(J)** microscopic view.

The evaluation of cell death was conducted using Evans Blue staining, a non-toxic, water-soluble dye widely used to specifically stain dead cells. Tissues infected with *P. aphanidermatum* exhibited intense blue staining, indicating a higher level of cell death in pathogen-treated samples (Figure 2). Among the treatment of fractions of fungal crude extracts, tissues treated with F3 showed a noticeable blue coloration, though less intense than the pathogen-only samples, while F2 and F1 treatments resulted in progressively lower staining intensities. Control plant samples, in contrast, remained unstained, indicating an absence of cell death, whereas the highest cell death was observed in the pathogen-infected tissues.

### 3.7 H₂O₂ accumulation in leaves visualized by DAB staining

**Figure 3:**
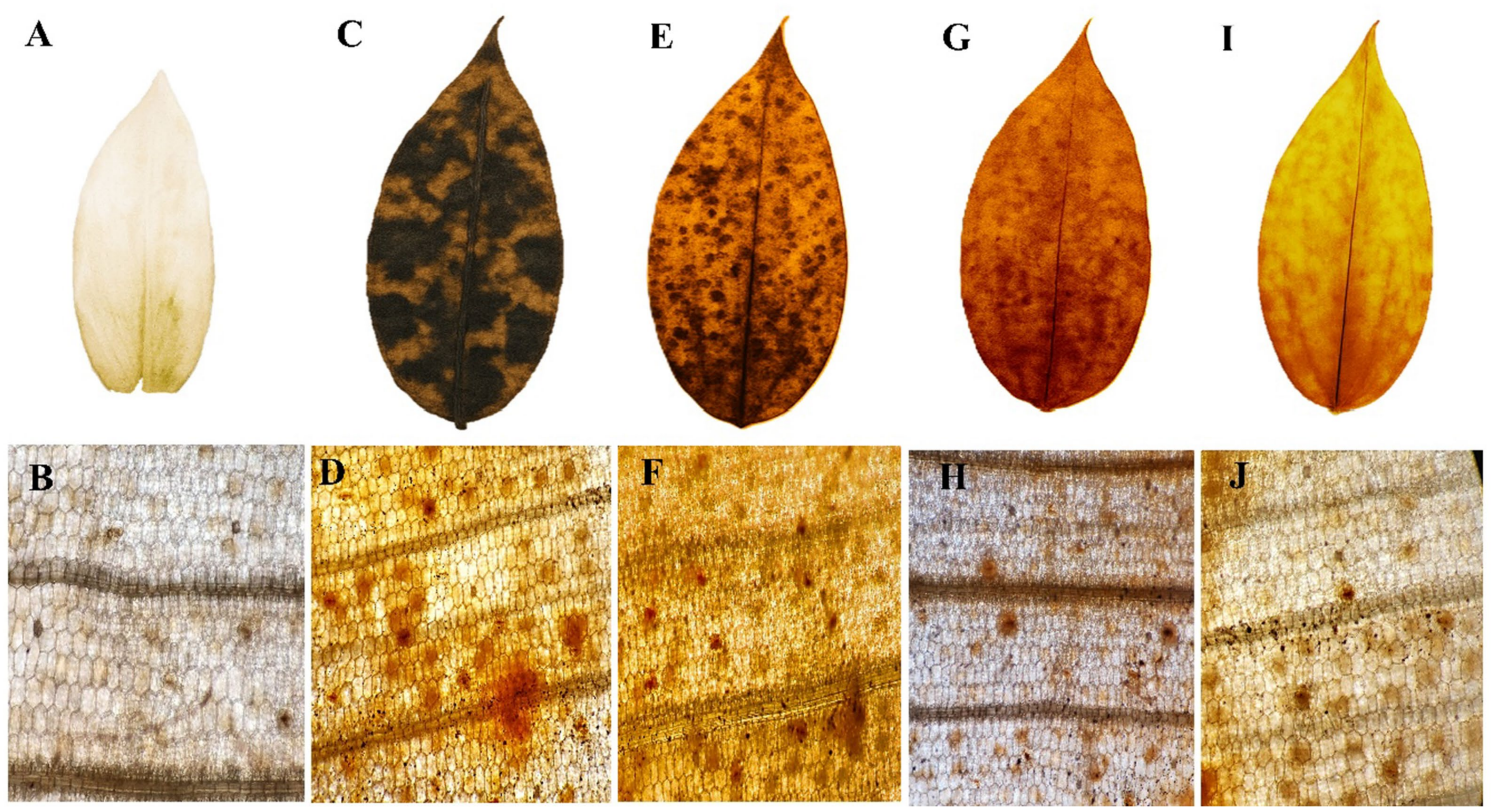
H_2_O_2_ accumulation in ginger leaves visualized by 3′3-diaminobenzidine (DAB) staining. **(A)** control leaf; **(B)** microscopic view; **(C)** *P. aphanidermatum* treated leaf; **(D)** microscopic view; **(E)** fraction 1 treated leaf; **(F)** microscopic view; **(G)** fraction 2 treated leaf; **(H)** microscopic view; **(I)** fraction 3 treated leaf; **(J)** microscopic view.

Hydrogen peroxide (H₂O₂) production was visualized as a reddish-brown stain using DAB staining, which appeared more prominently in the *Pythium* sp. infected plant samples (Figure 3). Quantitative analysis of H₂O₂ content was also conducted in ginger plants prior to and following pathogen infection, as well as after treatment with fungal crude extract fractions. Notable changes were observed in plants treated with fungal crude extract fractions, both before and after the pathogen challenge; however, the response was more pronounced and evident in the *Pythium* sp. infected plants.

### 3.8 Effect on chlorophyll content

**Figure 4:**
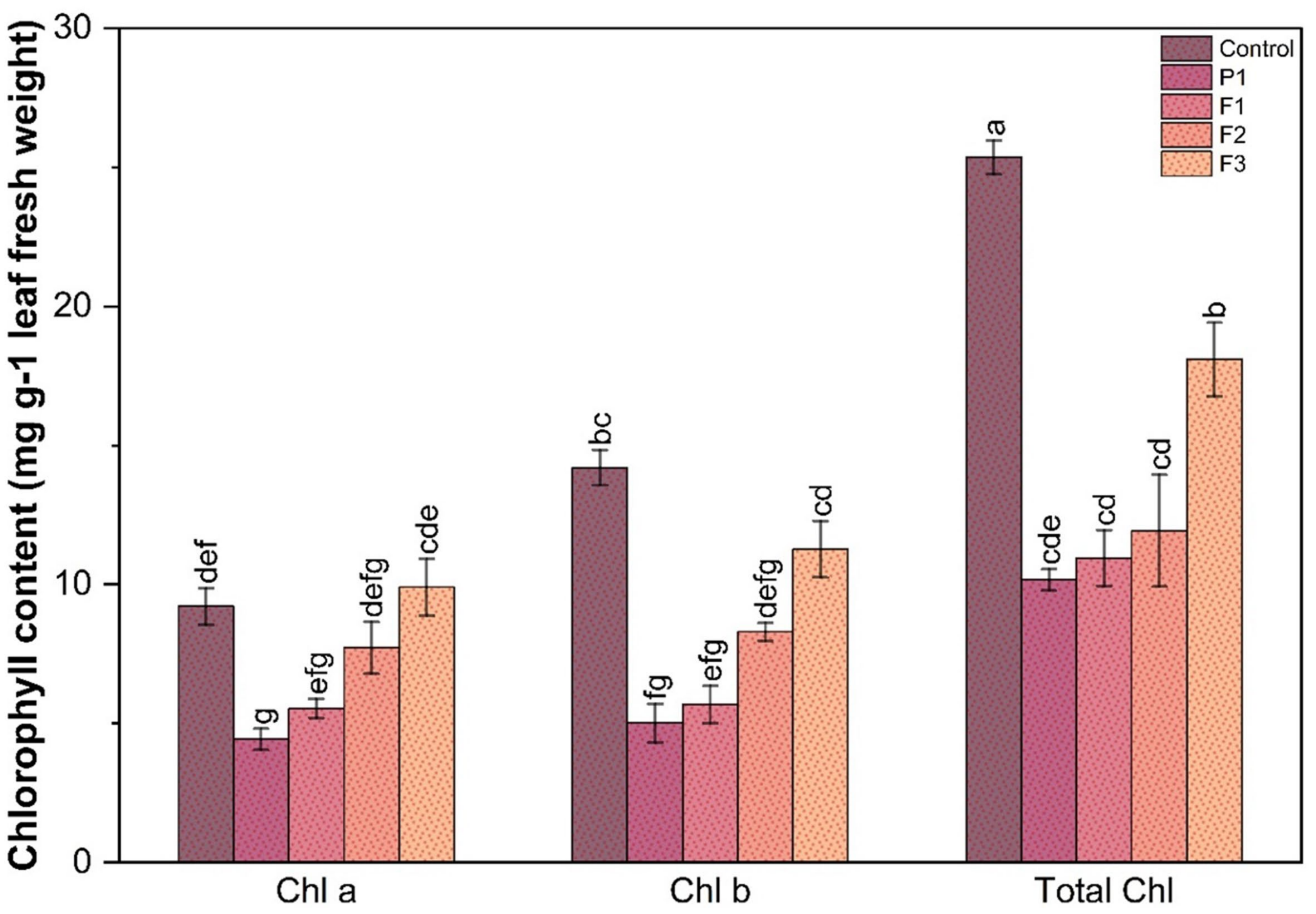
Effect on chlorophyll content in ginger plants infected with *P. aphanidermatum* and their extracted fractions 1, 2, 3.

The percentage contribution of Chl a, Chl b, and total Chl to the overall chlorophyll content in ginger plants under different treatments, including control, *P. aphanidermatum*, and fractions of fungal crude extracts of both pathogens, revealed distinct trends (Figure 4). Control plants exhibited the highest total chlorophyll content, with Chl a contributing approximately 65–70% and Chl b accounting for 30–35%, indicative of optimal photosynthetic pigment levels under non-stress conditions. *P. aphanidermatum* infected plants displayed a more severe impact, with the Chl a proportion further decreasing to 45–50% and Chl b increasing to 50–55%, highlighting greater chlorophyll degradation. Fractions of fungal crude extract, i.e., F1, F2, and F3 of *P. aphanidermatum* treatments showed varying degrees of recovery in chlorophyll content, with F3 being the most effective. In F3-treated plants, Chl a contributed approximately 60–65% and Chl b accounted for 35–40%, nearing the levels observed in control plants. F2-treated plants displayed slightly lower recovery, with Chl a contributing 55–60% and Chl b 40–45%, indicating moderate recovery. F1-treated plants exhibited the least recovery among the fractions, with Chl a contributing 50–55% and Chl b 45–50%. Overall, pathogen infections significantly reduced the proportion of Chl a while increasing the relative contribution of Chl b to the total chlorophyll pool. However, upon fungal crude extract treatments, particularly F3, of both pathogens restored the balance between Chl a and Chl b, thereby improving photosynthetic potential and alleviating pathogen-induced stress. The chlorophyll content is a critical indicator of photosynthetic efficiency and overall plant health. The significant decline in chlorophyll levels in plants infected by *P. aphanidermatum* reflects the impact of pathogen stress, which likely induces chlorophyll degradation or inhibits its biosynthesis. The differential response of ginger plants to *P. aphanidermatum* infections suggests variability in the pathogenicity or mode of action of the two pathogens. *P. aphanidermatum* more pronounced effect on chlorophyll levels indicates that it may employ more aggressive mechanisms, such as higher toxin production or stronger interference with the photosynthetic pathways.

### 3.9 Antioxidant enzyme activities

To assess the impact of fungal infections and treatments on oxidative stress regulation, various antioxidant enzyme activities were measured over a time course from 0 to 96 h. The results demonstrated significant changes in enzymatic activity following fungal infection and subsequent treatment interventions.

#### 3.9.1 Superoxide dismutase activity

**Figure 5:**
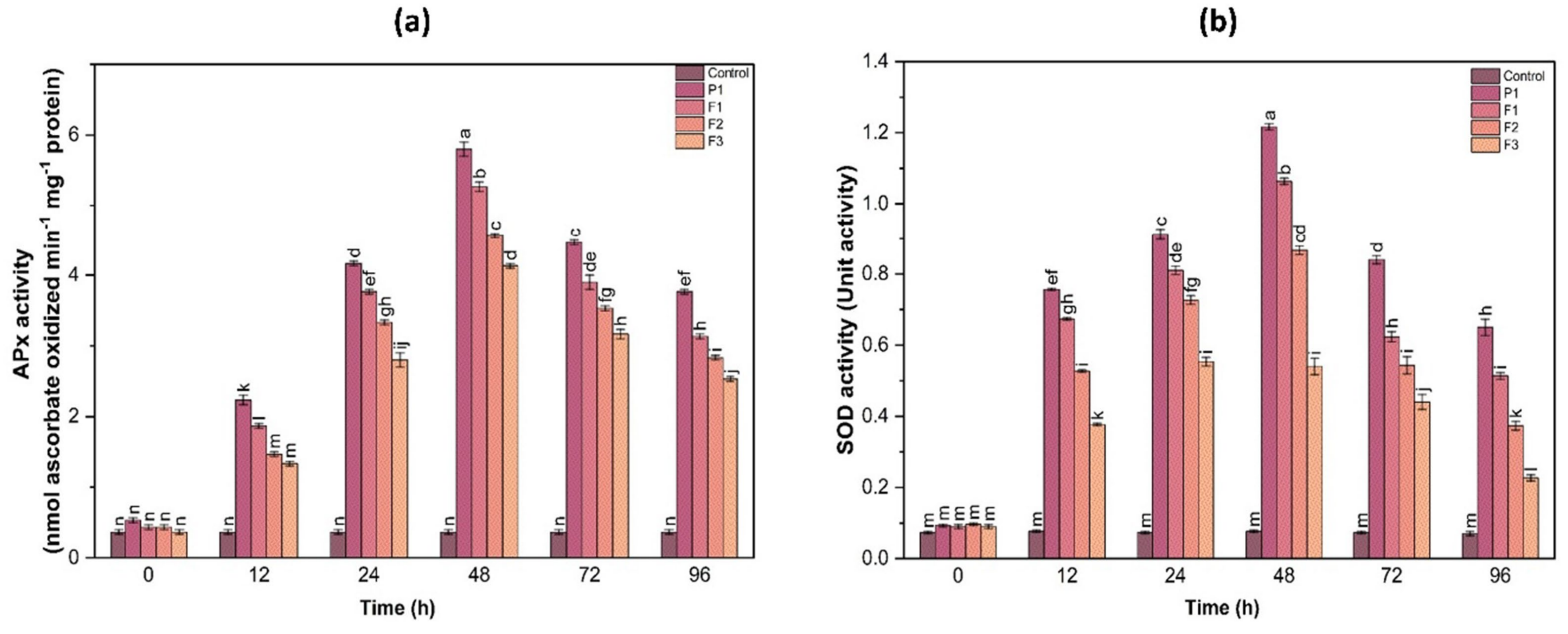
Ascorbate peroxidase (APx) activity **(a)** and superoxide dismutase (SOD) activity **(b)** in ginger plants at various time intervals following infection with *P. aphanidermatum* (P1), as well as after treatment with fungal crude extract fractions F1, F2, and F3. Control indicates untreated plants. Enzyme activity was measured at 0, 24, 48, 72, and 96 h post-treatment. Bars represent mean ± SD of three biological replicates (*n* = 3). Statistical significance was determined using one-way ANOVA with Tukey’s test; different letters indicate significant differences (*p* < 0.05).

SOD is the first line of defense against superoxide radicals, converting them into less harmful molecules. A significant increase in SOD activity was observed at 48 h, showing a 4.1-fold increase in F1-treated plants compared to the control. The enzyme activity gradually decreased at 72 and 96 h, suggesting that early induction of superoxide scavenging mechanisms plays a crucial role in managing oxidative stress caused by fungal infections. The rapid upregulation of SOD activity in treated plants suggests an effective enzymatic response that curbs ROS accumulation and limits cellular damage (Figure 5).

#### 3.9.2 Ascorbate peroxidase activity

APX activity plays a crucial role in scavenging reactive oxygen species (ROS) and mitigating oxidative stress in plants. The results indicated a notable increase in APX activity post-treatment, peaking at 48 h before gradually declining at 72 and 96 h. Compared to the control, the treated groups (F1, F2, F3) exhibited a 2.5- to 4.2-fold increase in APX activity at the 48 h time point. Among the treatments, F1 displayed the highest induction of APX activity, followed by F2 and F3. This suggests that treatment with F1 had the most pronounced effect on stimulating the plant’s antioxidative response to fungal infection, effectively reducing ROS-induced damage (Figure 5).

#### 3.9.3 Catalase activity

Similar to APX, catalase activity showed a significant increase, particularly between 24 and 48 h post-infection (Figure 5). The maximum catalase activity was observed at 48 h, where F1-treated plants exhibited a 3.8-fold increase compared to the control. A gradual decline was noted after 72 h, indicating a transient yet effective antioxidative response to fungal-induced stress. Untreated infected plants maintained consistently lower catalase activity, underscoring the role of treatment in enhancing enzymatic defense mechanisms.

#### 3.9.4 Glutathione reductase activity

**Figure 6:**
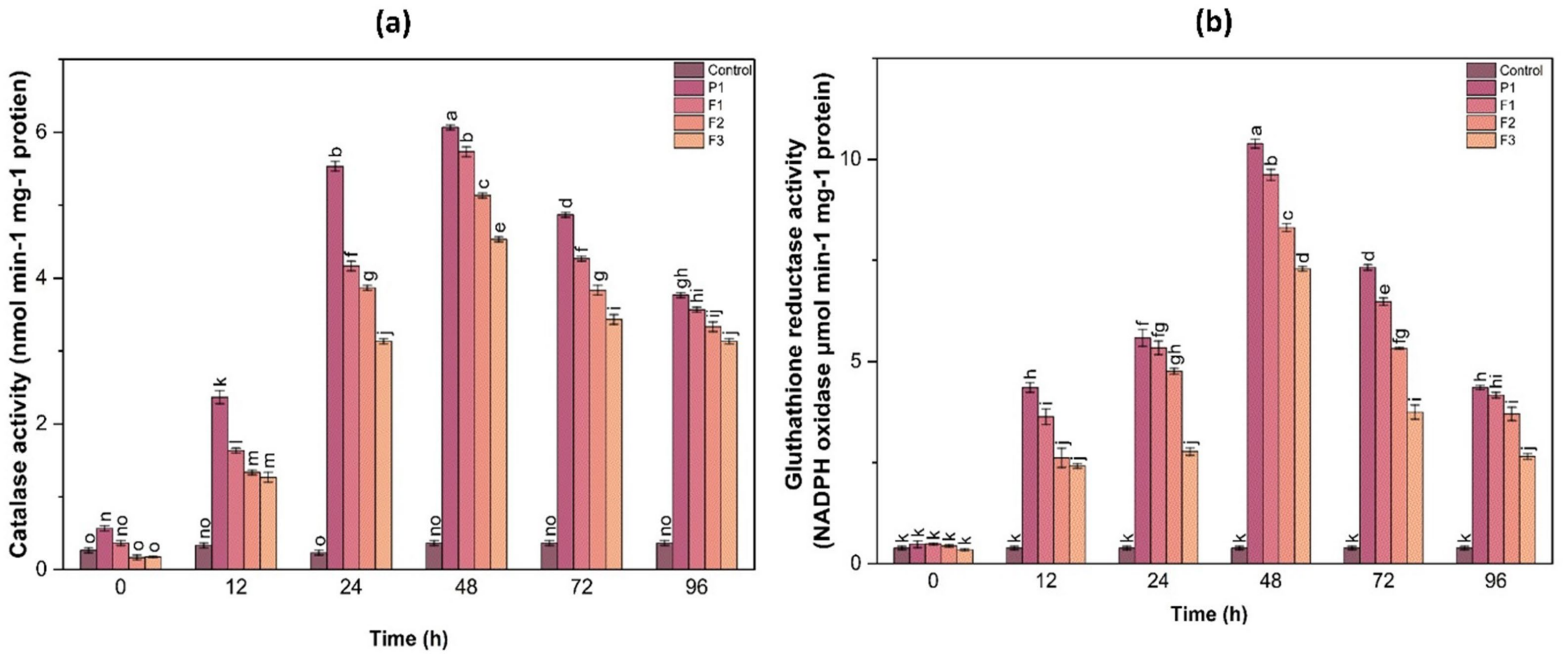
Catalase **(a)** and glutathione reductase **(b)** activities in ginger plants at different time intervals following infection with *P. aphanidermatum* (P1), as well as after treatment with fungal crude extract fractions F1, F2, and F3. Control represents untreated plants. Data are presented as mean ± SE. Different letters indicate statistically significant differences (*p* < 0.05) among treatments at each time point.

GR activity was another key parameter assessed in this study. The results indicated that GR activity peaked at 48 h in all treated samples, with F1-treated plants showing a 3.5-fold increase, while F3-treated plants exhibited a 2.8-fold increase compared to the control. The elevated GR activity highlights the importance of glutathione-mediated ROS detoxification, emphasizing the role of the applied treatments in reinforcing the plant’s antioxidant defense system (Figure 6).

### 3.10 Oxidative stress markers

To evaluate the extent of stress-induced damage, hydrogen peroxide (H₂O₂) production and lipid peroxidation (MDA content) were analyzed over time. These parameters serve as critical indicators of oxidative stress and membrane integrity in plant tissues.

#### 3.10.1 Hydrogen peroxide production

**Figure 7:**
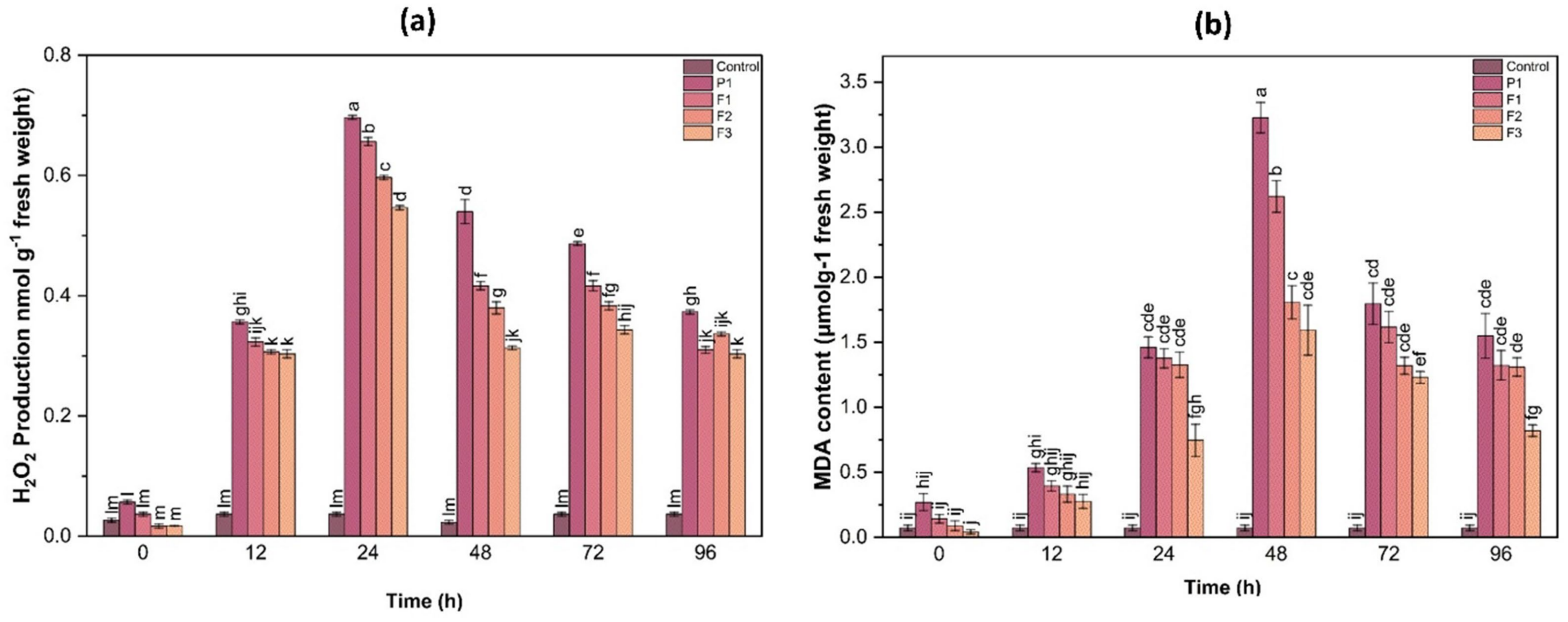
Hydrogen peroxide (H₂O₂) production **(a)** and malondialdehyde (MDA) content **(b)** in ginger plants at various time intervals after infection with *P. aphanidermatum* (P1) as well as following treatment with fungal crude extract fractions F1, F2, and F3. Control represents untreated plants. Data are expressed as mean ± SE. Different letters indicate statistically significant differences (*p* < 0.05) among treatments at each time point.

H₂O₂ is a major ROS molecule that can cause oxidative damage if not efficiently detoxified. Quantitative analysis revealed that H₂O₂ content in untreated infected plants peaked at 24 h post-infection, reaching 8.42 ± 0.36 μmol g^−1^ FW, which was a 2.8-fold increase compared to control plants (2.98 ± 0.22 μmol g^−1^ FW). In contrast, H₂O₂ levels in F1-, F2-, and F3-treated plants were 4.12 ± 0.31, 5.26 ± 0.29, and 5.88 ± 0.34 μmol g^−1^ FW, respectively, all significantly lower than the control (*p* < 0.05)However, treated plants (F1, F2, F3) exhibited significantly lower H₂O₂ levels, indicating the effectiveness of treatments in reducing ROS accumulation. The F1 treatment displayed the most substantial decline in H₂O₂ content, followed by F2 and F3, further validating its role in alleviating oxidative stress (Figure 7).

#### 3.10.2 Lipid peroxidation (MDA content)

Lipid peroxidation, quantified as MDA content, serves as an indicator of membrane damage caused by oxidative stress. Infected untreated plants showed the highest MDA accumulation (7.85 ± 0.41 nmol g^−1^ FW), indicating extensive membrane damage. F1-, F2-, and F3-treated plants showed significantly reduced MDA levels at 3.38 ± 0.27, 4.56 ± 0.30, and 5.11 ± 0.28 nmol g^−1^ FW, respectively (*p* < 0.05), corresponding to 2.3-, 1.7-, and 1.5-fold reductions compared to the control. This suggests that treatments helped mitigate oxidative membrane damage by enhancing the antioxidative defense system, thereby reducing lipid peroxidation (Figure 7).

### 3.11 Phenolic metabolism enzymes

In addition to antioxidant enzymes, this study assessed the activity of phenylalanine ammonia lyase (PAL) and polyphenol oxidase (PPO), which play crucial roles in plant defense mechanisms by synthesizing phenolic compounds and lignin.

#### 3.11.1 Phenylalanine ammonia-lyase activity

**Figure 8:**
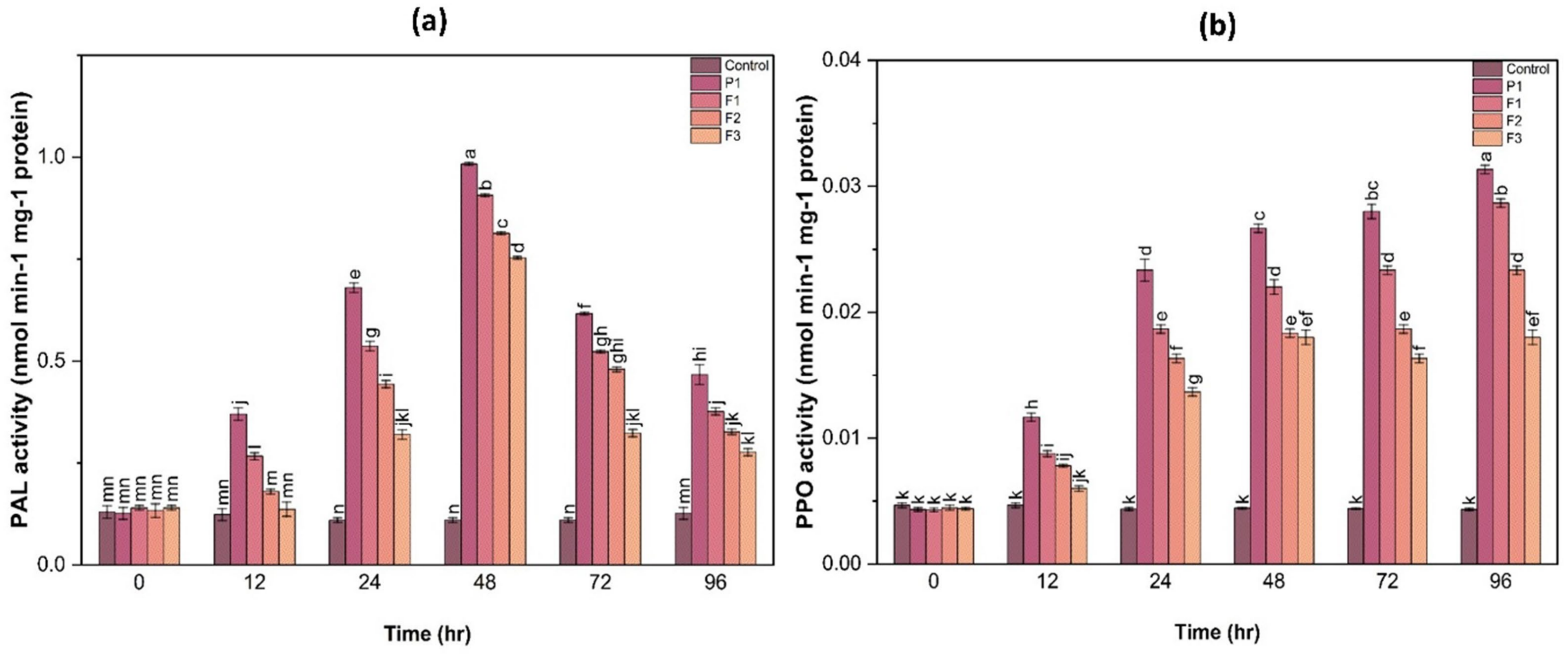
Phenylalanine ammonia-lyase (PAL) activity **(a)** and polyphenol oxidase (PPO) activity **(b)** in ginger plants at various time intervals after infection with *P. aphanidermatum* (P1) as well as following treatment with fungal crude extract fractions F1, F2, and F3. Control denotes untreated plants. Data are shown as mean ± SE. Different letters indicate statistically significant differences (*p* < 0.05) among treatments at each time point.

PAL activity was significantly higher in treated plants at 48 h post-infection. Among the treatments, F1-treated plants exhibited a 3.2-fold increase in PAL activity compared to control. This suggests that fungal infection triggered a defense response that was further enhanced by the treatments, leading to an increased production of phenolic compounds involved in disease resistance. The induction of PAL activity in treated samples highlights the role of secondary metabolites in strengthening plant defense against pathogen attack (Figure 8).

#### 3.11.2 Polyphenol oxidase activity

PPO activity followed a similar trend, with treated plants showing a 2.5- to 3.8-fold increase in PPO activity at 48 h compared to the control (Figure 8). The highest PPO activity was observed in F1-treated plants, followed by F2 and F3, suggesting enhanced lignification and structural reinforcement of plant tissues as a defense mechanism. The significant increase in PPO activity in treated samples indicates the potential role of phenolic metabolism in countering fungal infections.

The biochemical and enzymatic analyses confirmed that fungal infection led to severe oxidative stress, resulting in increased ROS production and lipid peroxidation. However, the application of treatments (F1, F2, F3) significantly ameliorated oxidative damage by enhancing the activity of key antioxidant enzymes such as APX, CAT, SOD, and GR. Among all treatments, F1 was the most effective, displaying the highest induction of enzymatic activities and the most substantial reduction in H₂O₂ and MDA levels. The chlorophyll content analysis further indicated that fungal infections compromised photosynthetic function, whereas treatments, particularly F1, significantly restored chlorophyll levels. The cell death assay using Evans blue staining provided additional evidence that fungal infection induced significant cell death, but treatment application reduced cellular damage and improved plant viability. Overall, the study highlights that fungal infection induces substantial oxidative stress in ginger plants, and provide insight into the pathogenic mechanisms of *P. aphanidermatum* and the phytotoxic potential of its metabolites. The study lays the groundwork for future research on detailed chemical characterization of bioactive fractions and the validation of these findings under natural field conditions. The findings emphasize the potential application of these treatments as sustainable strategies for managing fungal diseases in ginger cultivation.

### 3.12 Gas chromatography–mass spectrometry

**Figure 9:**
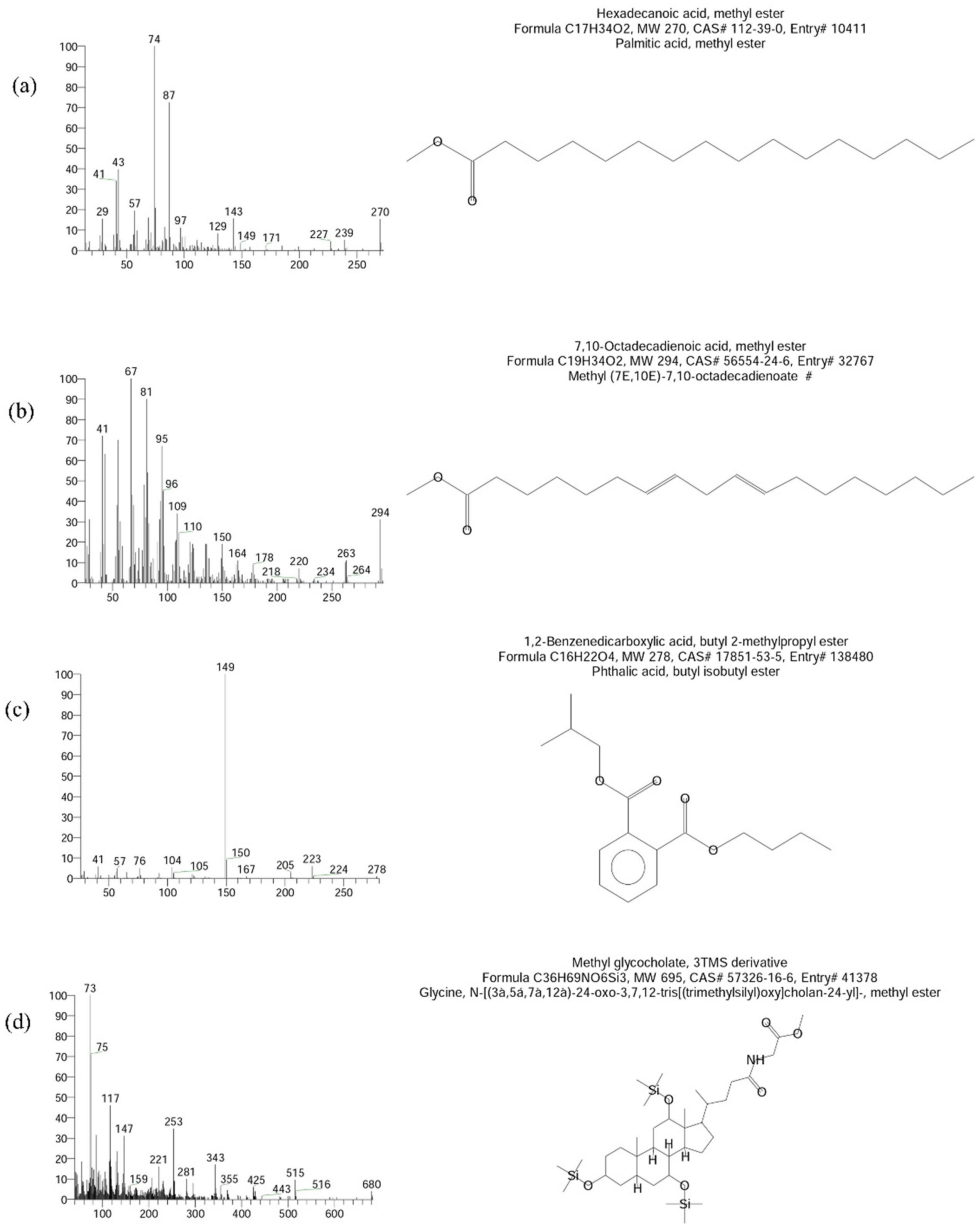
Mass spectrum and compound structure of major compounds present in the potential fraction 1 of fungal crude extract isolated from *Pythium aphanidermatum*. **(a–d)** represent the identified compounds.

The Gas Chromatography–Mass Spectrometry (GC–MS) analysis of the fungal extract revealed a rich and complex profile of bioactive secondary metabolites. More than fifty compounds were identified through spectral matching with the NIST and Wiley libraries, encompassing diverse chemical classes such as long-chain alkanes, alkenes, fatty acid esters, alcohols, aldehydes, terpenoids, steroids, phthalates, siloxanes, and nitrogen-containing compounds. One of the most prominent compounds detected was hexadecanoic acid, methyl ester (methyl palmitate), which exhibited the highest peak area (9.15%) at a retention time of 14.48 min. Other notable fatty acid esters included methyl stearate, methyl linolelaidate, methyl 10,13-octadecadiynoate, and various methylated octadecadienoic acids, all of which are known to contribute to antimicrobial and antioxidant activities. Hydrocarbon derivatives such as 2,6,10-trimethyl-tetradecane, 6-methyl-octadecane, and 1-chloro-octadecane were also detected, indicating the presence of non-polar, membrane-interacting metabolites. In addition, significant peaks corresponded to biologically active sesquiterpenes and terpenoids like ledol, globulol, and epiglobulol, which are recognized for their antimicrobial and anti-inflammatory properties. The detection of steroidal compounds such as betamethasone acetate and 3,9-epoxypregn-16-en-20-one further emphasized the chemical diversity and potential pharmaceutical relevance of the extract. Moreover, nitrogenous derivatives like phenethylamine and 3-benzyloxy-2-fluoro-*α*-hydroxy compounds were present, which may exhibit pharmacological or neuroactive effects. Several phthalate esters, including dibutyl phthalate and 1,2-benzenedicarboxylic acid butyl octyl ester, were identified, known for their antibacterial and anti-biofilm properties. Additionally, multiple cyclic siloxanes such as hexasiloxane, heptasiloxane, and octasiloxane were observed, possibly originating from analytical artifacts, yet frequently reported in fungal metabolomic profiles. Altogether, the GC–MS results highlight the metabolic versatility of the fungal isolate, reinforcing its potential as a source of pharmacologically significant compounds and justifying its observed antibacterial and antioxidant activities (Figure 9). While several compounds were identified from F1, Figure 9 displays only the four most abundant compounds based on GC–MS peak area, retention time, and spectral matching confidence. These were selected for emphasis due to their known bioactivity and possible roles in phytotoxicity. A complete list of detected compounds in F1 has been provided in Table 1.

**Table 1:** Chemical profile of the potential fraction 1of fungal crude extract isolated from *P. aphanidermatum.*

S. NO.	RT	Compound name	Area %	CAS number
1	10.73	Tetradecane, 2,6,10-trimethyl-	4.50	14905-56-7
2	11.87	2-Hexadecanol	1.71	14852-31-4
3	12.56	Globulol	2.80	51371-47-2
4	14.48	Hexadecanoic acid, methyl ester	9.15	112-39-0
5	14.94	1,2-Benzenedicarboxylic acid, butyl octyl ester	8.54	84-78-6
6	15.18	10,13-Octadecadiynoic acid, methyl ester	1.84	18202-24-9
7	16.23	7,10-Octadecadienoic acid, methyl ester	5.57	56554-24-6
8	16.51	Heptadecanoic acid, 9-methyl-, methyl ester	3.31	54934-57-5
9	22.84	3,9-Epoxypregn-16-en-20-one, 3-methoxy-7,11,18-triacetoxy-	1.89	NA
10	24.58	Hexasiloxane, 1,1,3,3,5,5,7,7,9,9,11,11-dodecamethyl-	4.63	995-82-4
11	24.90	Heptasiloxane, 1,1,3,3,5,5,7,7,9,9,11,11,13,13-tetradecamethyl-	1.94	19095-23-9
12	25.10	Octasiloxane, 1,1,3,3,5,5,7,7,9,9,11,11,13,13,15,15-hexadecamethyl-	3.06	19095-24-0
13	25.19	Pentasiloxane, 1,1,3,3,5,5,7,7,9,9-decamethyl-	5.52	995-83-5
14	25.32	Methyl glycocholate, 3TMS derivative	5.50	995-83-5
15	25.39	7,7,9,9,11,11-Hexamethyl-3,6,8,10,12,15-hexaoxa-7,9,11-trisilaheptadecane	2.30	NA
16	25.44	6,6,8,8,10,10-Hexamethyl-2,5,7,9,11,14-hexaoxa-6,8,10-trisilapentadecane	2.68	NA
17	25.61	Octasiloxane, 1,1,3,3,5,5,7,7,9,9,11,11,13,13,15,15-hexadecamethyl-	2.62	19095-24-0
18	25.76	Methyl glycocholate, 3TMS derivative	1.70	57326-16-6
19	26.08	Methyl glycocholate, 3TMS derivative	2.80	57326-16-6
20	26.49	Octasiloxane, 1,1,3,3,5,5,7,7,9,9,11,11,13,13,15,15-hexadecamethyl-	2.52	19095-24-0
21	26.55	Octasiloxane, 1,1,3,3,5,5,7,7,9,9,11,11,13,13,15,15-hexadecamethyl-	2.03	19095-24-0
22	26.90	Heptasiloxane, 1,1,3,3,5,5,7,7,9,9,11,11,13,13-tetradecamethyl-	2.76	19095-23-9
23	27.51	Heptasiloxane, 1,1,3,3,5,5,7,7,9,9,11,11,13,13-tetradecamethyl-	2.79	19095-23-9
24	28.42	Hexasiloxane, 1,1,3,3,5,5,7,7,9,9,11,11-dodecamethyl-	3.09	995-82-4
25	29.62	Hexasiloxane, 1,1,3,3,5,5,7,7,9,9,11,11-dodecamethyl-	1.67	995-82-4
26	31.59	Pentasiloxane, 1,1,3,3,5,5,7,7,9,9-decamethyl-	3.85	995-83-5
27	33.88	Methyl glycocholate, 3TMS derivative	1.97	995-83-5

## 4 Discussion

Ginger is an economically valuable crop, widely used for its medicinal, nutritional, and culinary properties. However, fungal infections pose a major threat to ginger production, leading to severe economic losses. Pathogens such as *P. aphanidermatum* are particularly notorious for causing extensive tissue necrosis, root rot, and wilt, severely impacting ginger cultivation worldwide. These pathogens not only invade the host tissues but also induce substantial biochemical and physiological alterations within the plant (Hendrix and Campbell, 1973; Jiménez-Gasco et al., 2004). Recent studies have emphasized the complexity of the ginger–*Pythium* interaction. For instance, Sheikh et al. (2023a,b) demonstrated that volatile organic compounds (VOCs) produced by *Pythium oligandrum* promote ginger growth and systemic resistance, highlighting the dual role of some *Pythium* species as biocontrol agents. Moreover, *P. oligandrum* was shown to parasitize *P. myriotylum*, a virulent pathogen frequently recovered from soft rot-infected ginger rhizomes in China (Meena et al., 2015; Daly et al., 2022). These findings underscore the relevance of pathogen–antagonist interactions and support the need for characterizing virulence mechanisms and toxic metabolites in *P. aphanidermatum* as presented in the current study. By integrating biochemical and physiological analyses with gas chromatography–mass spectrometry (GC–MS) analysis, this study provides a comprehensive evaluation of the complex interactions between ginger plants and pathogenic fungi. This study significantly advances the understanding of plant responses to fungal pathogens, specifically *P. aphanidermatum*, affecting ginger (*Zingiber officinale*). In this study, amplification and sequencing of the internal transcribed spacer (ITS) regions using ITS1 and ITS4 primers confirmed the identity of *P. aphanidermatum*. This molecular approach demonstrated superiority over traditional methods, consistent with previous research (Schoch et al., 2012), where similar techniques successfully identified pathogenic fungi in economically important crops such as tomato, banana, wheat, and maize (Chitwood-Brown et al., 2021). Pathogenicity assessments revealed the aggressive nature of these fungal pathogens, with infected ginger plants exhibiting severe physiological stress, including chlorosis, necrosis, and extensive rhizome rot. Additionally, research on *Pythium* infections in cucumbers showed that pathogen presence increases root rot and disrupts nutrient uptake, leading to chlorosis, similar to what was observed in ginger plants (Hendrix and Campbell, 1973; Coque et al., 2020). These findings highlight the importance of environmental management strategies, including improved drainage and reduced water stagnation, in mitigating fungal disease severity, as emphasized in similar studies on tomato (Szczechura et al., 2013). The current study elucidates the pathogenic potential and phytotoxic effects of *P. aphanidermatum*, a well-known oomycete pathogen implicated in ginger soft rot and rhizome decay. Pathogenicity testing via wound inoculation and detached leaf assays revealed rapid symptom development, including foliar wilting, chlorosis, and rhizome rot within 14 days post-inoculation. These symptoms align with previous findings that emphasize the virulent behavior of *P. aphanidermatum* on ginger and other monocot hosts under conducive environmental conditions (El Jarroudi et al., 2011). The confirmation of its pathogenicity in both whole plants and leaf tissue assays underscores its destructive potential and the need for in-depth toxicological characterization of its secreted metabolites. Column chromatography of the crude culture filtrate led to the isolation of ten fractions (F1–F10), which were independently assessed for their phytotoxic effects using a tomato leaf necrosis assay. The results revealed significant inter-fraction variability in terms of tissue damage, indicative of differential biochemical and biophysical impacts. Notably, fraction 1 (F1) emerged as the most toxic, consistently producing the highest percentage of necrotic leaf area across all time points (Day 1 to Day 5), peaking at approximately 96% necrosis, compared to moderate and low responses in fractions F2–F6 and F7–F10, respectively. This suggests that F1 is enriched in potent phytotoxins capable of causing irreversible cellular damage and possibly mimicking or enhancing the virulence observed during in planta infections (Hoerger et al., 2009). In addition to pathogen-inoculated plants (*P. aphanidermatum*), plants treated with fungal crude extract fractions (F1, F2, F3) were analyzed to assess the impact of different fungal metabolites on disease progression. Among these, F1 treatment exhibited the maximum pathogenicity effect for *P. aphanidermatum*, though not exceeding the direct pathogenic effect of *P. aphanidermatum* itself. This suggests that F1 contained high concentrations of pathogenic metabolites that contributed significantly to stress induction and oxidative damage, similar to findings in wheat infected by *Fusarium graminearum*, where fungal toxin deoxynivalenol (DON) induced severe oxidative damage and physiological stress (Audenaert et al., 2013). However, F2 and F3 treatments caused comparatively milder symptoms, resembling results from studies on maize, where certain fungal metabolites acted as growth regulators rather than pure toxins (Bryła et al., 2022; Cheng et al., 2009). Although the bioactivity of fractions F1–F3 was clearly established based on physiological and biochemical assays, their specific chemical composition remains largely unresolved, except for F1, which was subjected to GC–MS analysis. Identification of active metabolites in F2 and F3 will require further structural elucidation using advanced tools such as NMR or LC–MS/MS. Additionally, bioassay-guided fractionation would help to isolate and confirm compound-specific phytotoxicity.

The Evans blue staining assay effectively demonstrated the extent of cellular death induced by pathogen treatments and different fractions. The intensity of blue staining directly correlated with cellular injury severity. *P. aphanidermatum* treatment resulted in the highest level of cell death, suggesting greater pathogenicity or cytotoxic potential. This observation aligns with microscopic evidence showing increased cellular damage and dye uptake. Comparatively, similar research by Singh et al. (2019) and Gao et al. (2020) utilized Evans blue staining to evaluate pathogen-induced cellular damage in plant leaves, reporting consistent correlations between dye uptake and cell membrane integrity. Our findings are congruent with these studies, further validating Evans blue as a reliable method for quantifying cell death in plant pathology research. The relatively lower staining intensity seen in fraction 2 indicates minimal cytotoxic activity, potentially due to lower concentrations of active compounds or presence of less toxic constituents, paralleling observations by Zhou et al. (2021), who reported varying bioactivity across pathogen-derived fractions.

The observed H₂O₂ accumulation through DAB staining further supports oxidative stress as a significant response mechanism upon pathogen infection. This aligns with previous studies by Mittler (2017) and Liu et al. (2020), who reported increased H₂O₂ levels in plants upon pathogen attack, highlighting its role in plant defense signaling. Similarly, our findings corroborate with results by Wang et al. (2022), who demonstrated differential oxidative responses among pathogen-derived fractions, linking higher oxidative stress with greater pathogen virulence. The moderate oxidative stress observed in fraction 1 and fraction 3-treated leaves might indicate an intermediate response to pathogen-derived metabolites, consistent with findings from earlier studies by Xu et al. (2018).

These results provide valuable insights into the differential cytotoxic effects of pathogens and their fractions on leaf tissues, emphasizing the potential for fraction-specific activities. Further studies may involve biochemical studies of fractions and fungal pathogen-treated plants to identify active component responsible for observed cytotoxicity, potentially aiding in developing targeted plant protection strategies. The physiological responses of ginger plants varied significantly across treatments. *P. aphanidermatum* treatments resulted in severe chlorosis, necrosis, and rhizome deterioration, whereas plants treated with fungal crude extract fractions displayed differential responses. F1-treated plants exhibited substantial oxidative stress, leading to reduced chlorophyll content and higher levels of reactive oxygen species (ROS), lipid peroxidation, and membrane damage (Miller et al., 2010). In contrast, F2 and F3 treatments induced milder symptoms, with F3-treated plants demonstrating comparatively better chlorophyll retention and reduced oxidative damage, indicating potential protective effects or a less aggressive interaction with the plant. While the biological effects of fractions F1–F3 on oxidative stress and chlorophyll degradation were significant, the chemical identities of compounds in F2 and F3 remain uncharacterized. Only F1 was subjected to GC–MS analysis, revealing several dominant metabolites likely involved in stress induction (Kumar et al., 2018). However, the absence of comprehensive chemical profiling for F2 and F3 limits a precise understanding of their mechanism of action. Future studies should employ LC–MS/MS or NMR for detailed metabolite identification across all fractions, and bioassay-guided purification could help correlate specific compounds with physiological outcomes.

Similar reductions in chlorophyll content due to fungal infections have been reported in tomato and wheat plants infected by *Fusarium* species, where pathogen-induced oxidative stress significantly impairs photosynthetic efficiency (Ali et al., 2020; Matthews et al., 2023). In comparison, studies on rice infected by *Magnaporthe oryzae* found that fungal toxins specifically target chlorophyll synthesis pathways, mirroring the chlorophyll degradation seen in ginger plants (Sharma et al., 2012; Kawano et al., 2018). Cell death assays using Evans blue staining confirmed extensive cellular damage in infected plants. However, treatments significantly reduced cell death, with F1 showing the most protective effects. These findings underscore the potential of fungal crude extracts as sustainable strategies for disease management in ginger cultivation. In comparison, studies on rice infected by *Magnaporthe oryzae* found that fungal toxins specifically target chlorophyll synthesis pathways, mirroring the chlorophyll degradation seen in ginger plants (Sharma et al., 2012). Phenylalanine ammonia-lyase (PAL) and polyphenol oxidase (PPO) play crucial roles in synthesizing phenolic compounds and reinforcing plant defense. Both enzymes exhibited significant induction post-infection, with the highest activity observed at 48 h (Geethu et al., 2013). *P. aphanidermatum* treatments triggered the strongest PAL activity, aligning with increased oxidative stress markers. F3 consistently displayed the lowest PAL activity, suggesting a less stress-inducing or possibly mutualistic interaction. Similar responses were observed in soybean infected by *Phytophthora sojae*, where PAL and PPO upregulation played a crucial role in resistance (Graham and Graham, 1996).

In contrast, F3 consistently displayed the lowest PAL activity, suggesting a less stress-inducing or possibly mutualistic interaction, as seen in maize plants treated with *Trichoderma harzianum*, which promoted plant growth and provided resistance without excessive stress activation (Mastouri et al., 2012). PPO activity steadily increased over time, indicating a prolonged defense response. Similar to what has been observed in wheat infected with *Puccinia triticina*, where PPO activity correlated with pathogen resistance (Mandal et al., 2009; Wan et al., 2019). Moderate PPO induction in F1 and F2 suggests a balanced interaction, while F3 exhibited the lowest activity, reinforcing its potential as a beneficial endophyte, as seen in studies where beneficial fungi reduced stress-induced PPO activity in host plants (Harman et al., 2004).

Further work is needed to chemically characterize all fractions (F1–F10) to identify the full spectrum of metabolites responsible for bioactivity. Techniques such as LC–MS/MS or NMR would enable structural elucidation and confirm the mode of action of key metabolites. Moreover, bioassay-guided fractionation and metabolomic correlation with phytotoxic indices could help isolate lead compounds for potential antifungal or herbicidal applications. Gas chromatography–mass spectrometry (GC–MS) analysis provided insights into the fungal metabolites responsible for pathogenicity and plant stress responses. The analysis of F1 extracts revealed the presence of several bioactive compounds, including hexanoic acid and limonene, which have been implicated in pathogen virulence and oxidative stress induction. Although the exact molecular mechanism by which metabolites in fraction 1 induce oxidative stress was not directly studied, several identified compounds such as hexadecanoic acid methyl ester and phytol derivatives are known to impair membrane integrity and interfere with the antioxidant defense system of host cells (Kozieł et al., 2024). These effects may lead to overproduction of reactive oxygen species (ROS), lipid peroxidation, and subsequent cellular damage. Additionally, terpenoid compounds are reported to modulate signaling pathways and stress response genes in plants (Torres et al., 2006).

Further transcriptomic or proteomic analyses are warranted to confirm whether these metabolites interfere with key defense-related genes such as peroxidases, MAP kinases, or glutathione-related enzymes. Similar studies on *Fusarium* species infecting wheat reported the presence of trichothecenes, which disrupt cellular membranes and induce oxidative stress (Walter et al., 2010; Niehaus et al., 2014, 2017; Sharma et al., 2002). These compounds contribute to the observed physiological and biochemical disruptions in infected ginger plants, mirroring studies in tomato plants where certain secondary metabolites conferred resistance against *Botrytis cinerea* (Alfonso et al., 2019). The differential metabolite profiles across treatments further highlight the complex interplay between fungal metabolites and plant defense mechanisms. This study provides valuable insights into the molecular identification, pathogenicity, and biochemical responses of ginger plants to *P. aphanidermatum* infections. The findings emphasize oxidative stress as a central factor in disease progression and highlight the importance of antioxidant enzyme activation in plant defense. The identification of key fungal metabolites, such as hexanoic acid and limonene, further enhances our understanding of pathogen virulence strategies. Our study highlights the pathogen’s ability to induce severe oxidative stress and reveals the presence of potent phytotoxic metabolites (Aly et al., 2015).

These findings suggest new avenues for disease management: (i) use of ROS-scavenging compounds or biostimulants to mitigate oxidative damage, (ii) development of targeted fungicides or biocontrol agents that disrupt toxin biosynthesis, and (iii) deployment of antioxidant enzyme activity and chlorophyll degradation markers as early indicators of infection severity. Future work should integrate transcriptomic analysis to identify host defense gene expression under fungal stress, and metabolite-based screening of antifungal agents from natural sources (Gong et al., 2023; Bryła et al., 2022). Overall, the results support the integration of fungal crude extract treatments, particularly F1, into disease management strategies. Future research should focus on elucidating the molecular mechanisms underlying F1-induced defense responses and exploring its application in large-scale ginger cultivation. Sustainable biocontrol approaches involving non-pathogenic *Pythium* strains with antagonistic properties have also been explored as alternatives to chemical fungicides. For instance, *Pythium oligandrum* has shown potential in suppressing pathogenic *Pythium* spp. through mycoparasitism and metabolite antagonism (Sheikh et al., 2023a,b). While the current study did not evaluate biocontrol, our findings provide a complementary perspective by characterizing the biochemical impact of pathogen-derived fractions, offering a new direction for early intervention strategies. Additionally, integrating biochemical markers into routine disease surveillance could enhance early pathogen detection and improve disease management strategies. The multidisciplinary approach employed in this study serves as a model for similar research in other crop systems, contributing to sustainable agricultural practices and food security.

## 5 Conclusion

In conclusion, the study demonstrated that *P. aphanidermatum* induces oxidative stress in ginger plants, and the associated metabolites particularly those in fraction 1 may play a critical role in pathogenicity through ROS generation and membrane damage. The findings suggest that biochemical markers such as elevated H₂O₂ and MDA levels could be used for early detection of infection in ginger crops. Moreover, the bioactive metabolites identified here hold promise for developing natural fungicidal agents or stress-mitigating formulations, although further research is required to validate their efficacy. Integration of this knowledge into integrated disease management (IDM) strategies including the use of antagonistic biocontrol agents like *Pythium oligandrum* could improve disease resistance and reduce dependence on chemical fungicides. Limitations of the current study include the lack of complete chemical characterization of all active fractions (F2 and F3), and the absence of field-level testing under real agricultural conditions. Future work should focus on detailed metabolite profiling using NMR/LC–MS, bioassay-guided purification, and large-scale validation of findings under natural environmental setups.

## Data Availability

The datasets presented in this study can be found in online repositories. The names of the repository/repositories and accession number(s) can be found at: https://www.ncbi.nlm.nih.gov/, OP394047.1.
