# Supplementary material for: Molecular identification and pathogenic impact of Pythium aphanidermatum on ginger (Zingiber officinale): insights into oxidative stress, antioxidant responses, and mycotoxin profiling
Source: Front Microbiol. 2025 Sep 25;16:1626700. doi: 10.3389/fmicb.2025.1626700 (PMC12507739; doi:10.3389/fmicb.2025.1626700)
Supplement: Supplementary file 1 [file Table_1.DOCX]

**Molecular Identification and Pathogenic Impact of Pythium aphanidermatum on Ginger (Zingiber officinale): Insights into Oxidative Stress, Antioxidant Responses, and Mycotoxin Profiling**

Mukesh Meena^1,*^, Garima Yadav^1^, Priyankaraj Sonigra^1^, Tushar Mehta^1^, Adhishree Nagda^1^, Abhishek Sahoo^1^, and Prashant Swapnil^2^

^1^Laboratory of Phytopathology and Microbial Biotechnology, Department of Botany, Mohanlal Sukhadia University, Udaipur – 313001, Rajasthan, India

^2^Department of Botany, School of Basic Sciences, Central University of Punjab, Bathinda – 151401, India

Corresponding Author*:

**Mukesh Meena**

Email: mukeshmeenamlsu@gmail.com / drmukeshmeena321@mlsu.ac.in

Orcid Id: https://orcid.org/0000-0002-6336-1140


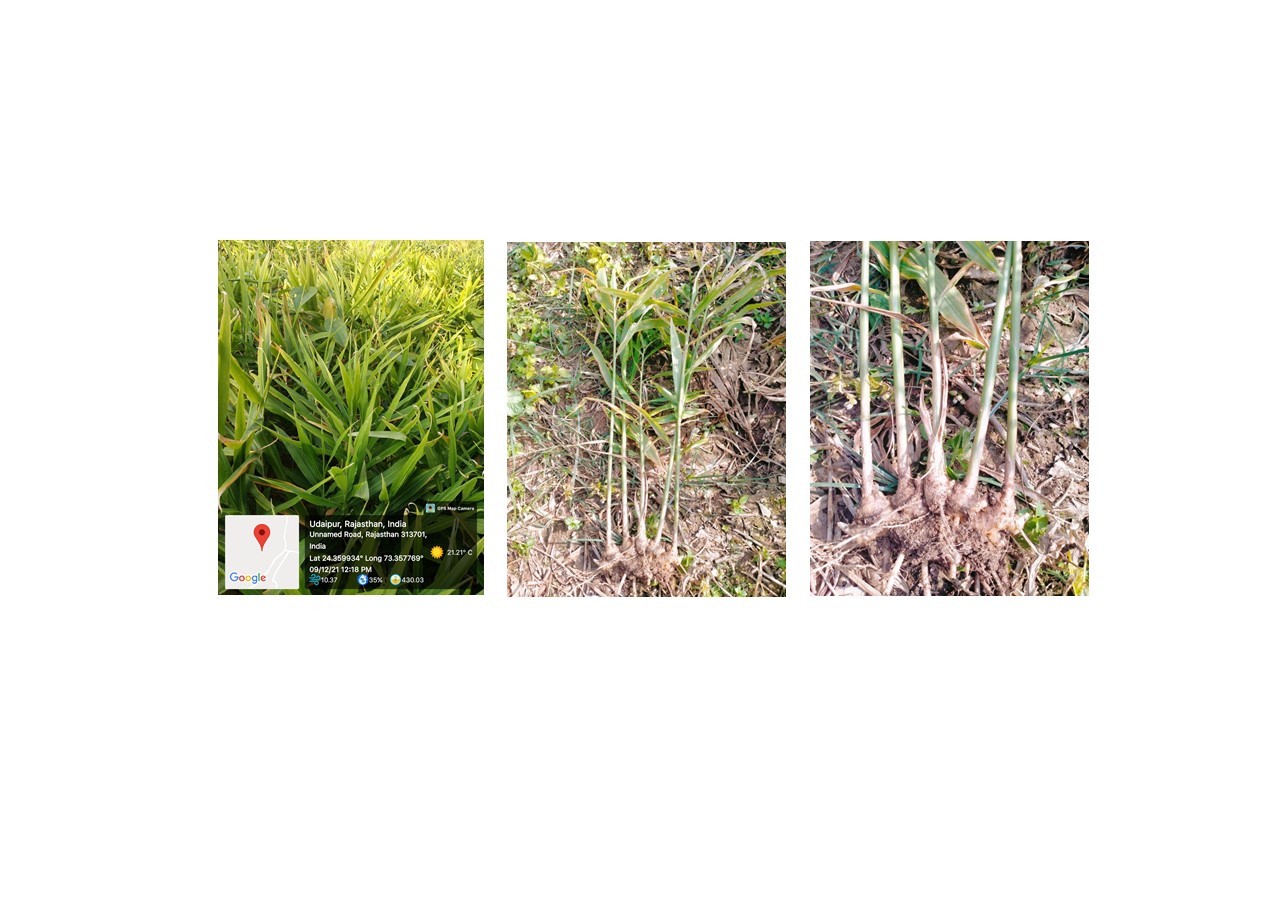


**Supplementary Figure 1:** Survey of ginger plant cultivation fields and collection sites. Pictures show the disease symptoms on ginger plants' leaves, stems and tubers.


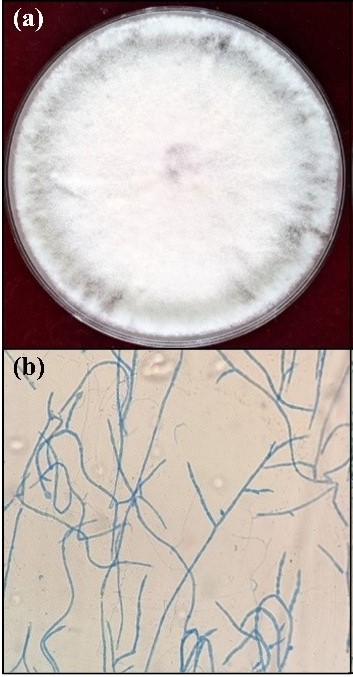


**Supplementary Figure 2:** Pathogen isolation from infected ginger on PDA. (a) *Pythium aphanidermatum* colony; (b) *Pythium aphanidermatum* hyphae under microscope.


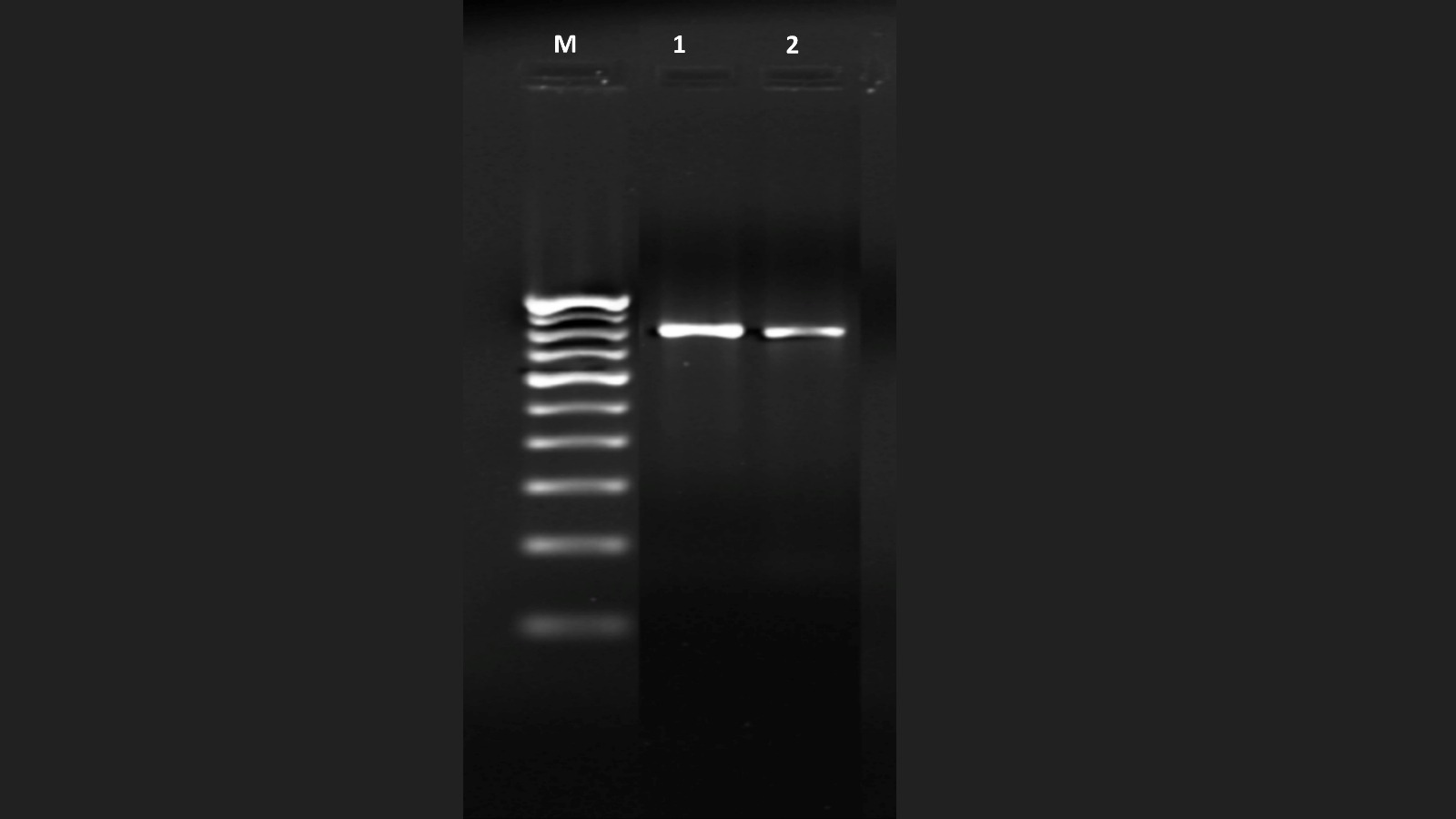


**Supplementary Figure 3:** Gel electrophoresis of PCR products with primers ITS1/ITS4 of DNA from fungal isolates. Lane M, molecular weight markers (1 kb ladder); lane 1 of *P. aphanidermatum*.


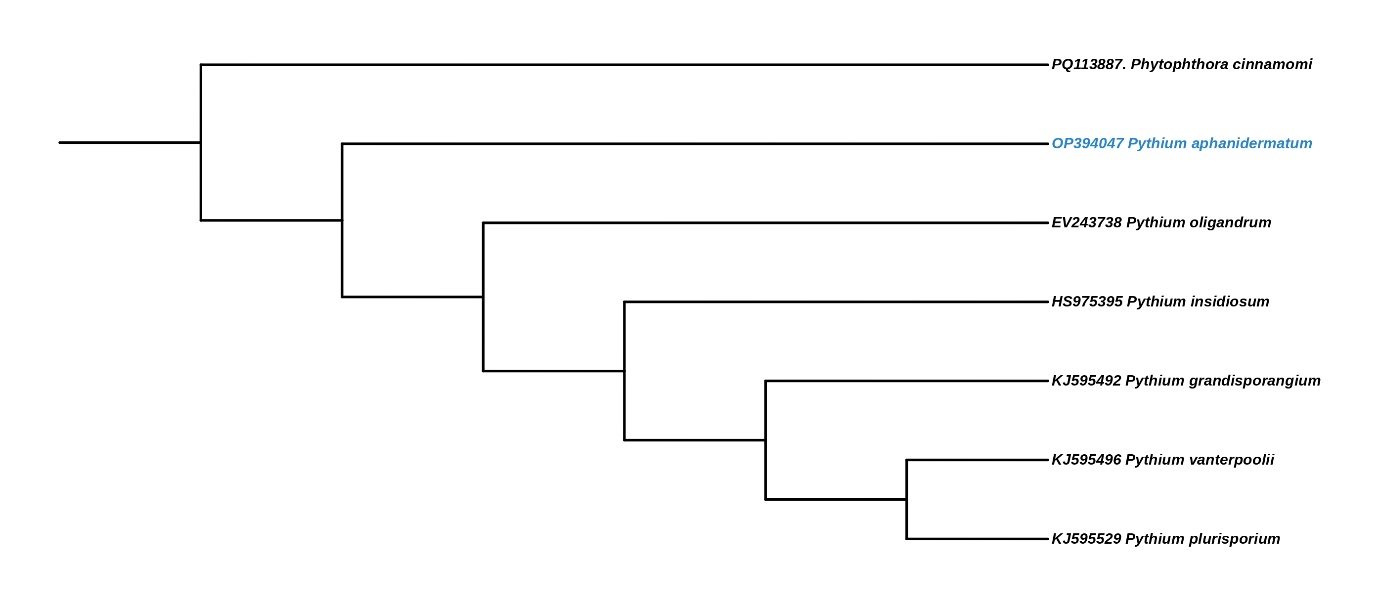


**Supplementary Figure 4:** Phylogenetic tree constructed based on ITS region sequences using the Neighbor-Joining method. The tree includes Pythium aphanidermatum (OP394047) and reference sequences retrieved from GenBank. Bootstrap values >50% (from 1000 replicates) are shown at nodes. The analysis indicates a close evolutionary relationship between P. aphanidermatum and P. insidiosum, with moderate bootstrap support (65%), validating the identity of the isolate used in this study.


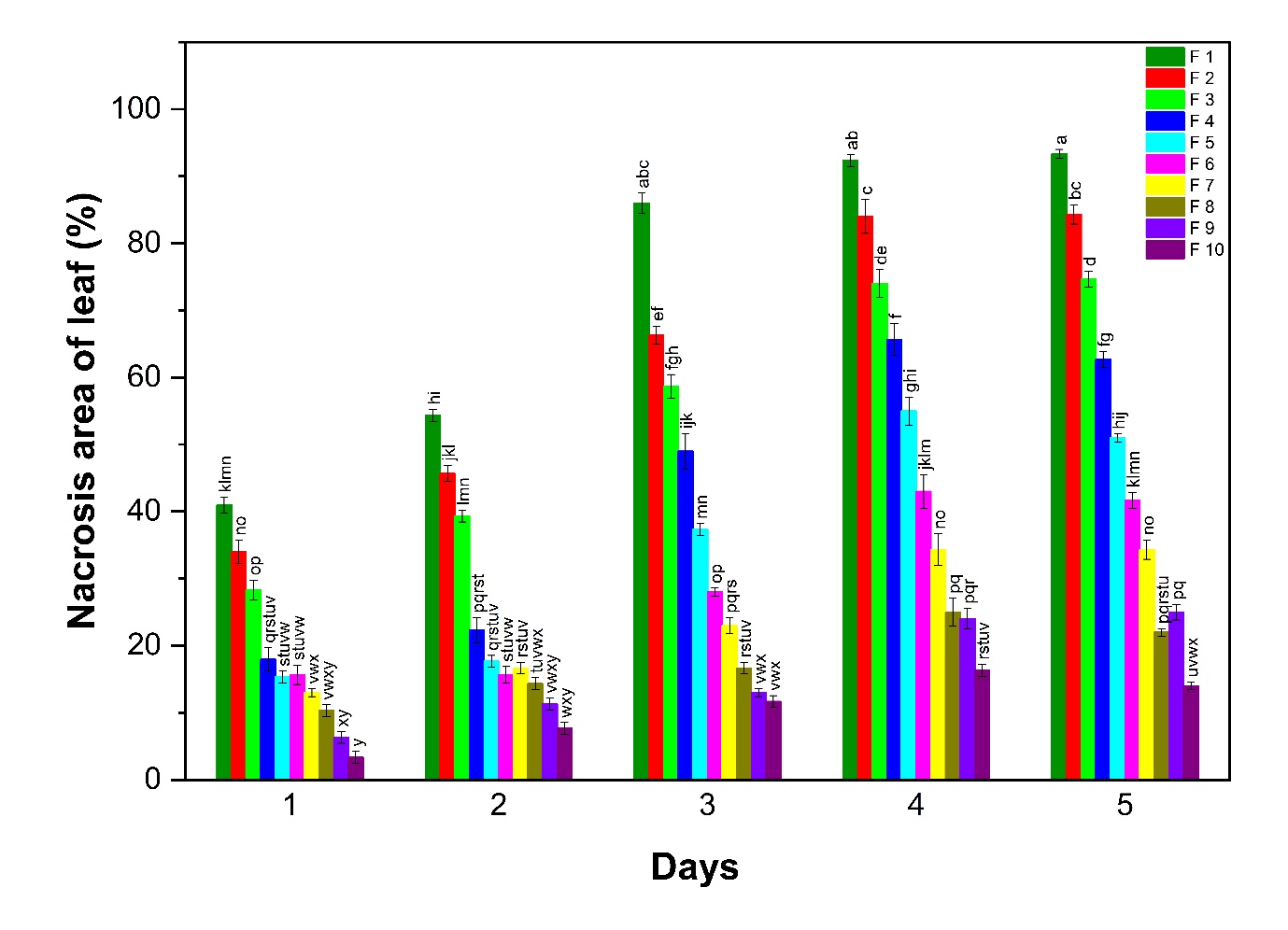


**Supplementary Figure 5:** Percentage necrotic area on ginger leaves treated with different toxin fractions (F1–F10) over 5 days. Bars represent mean ± SE; different letters indicate statistically significant differences (Tukey’s HSD, p < 0.05).


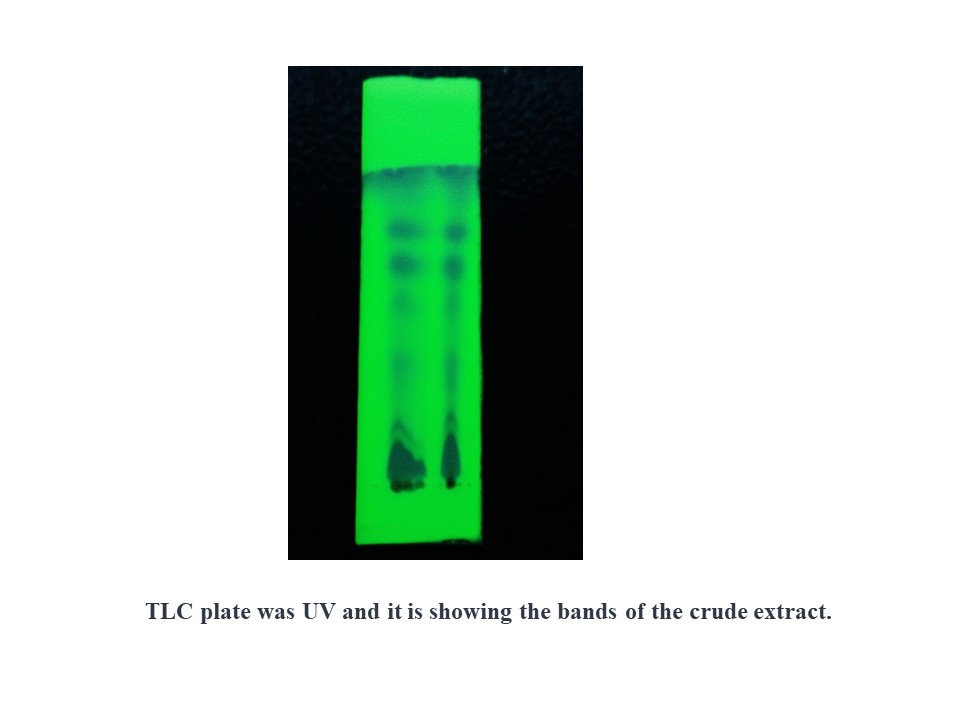


**Supplementary Figure 6:** The TLC plate under UV light shows the bands of the crude extract.
